# Primary multistep phosphorelay activation comprises both cytokinin and abiotic stress responses: insights from comparative analysis of *Brassica* type-A response regulators

**DOI:** 10.1093/jxb/erae335

**Published:** 2024-08-22

**Authors:** Katrina Leslie Nicolas Mala, Jan Skalak, Elena Zemlyanskaya, Vladislav Dolgikh, Veronika Jedlickova, Helene S Robert, Lenka Havlickova, Klara Panzarova, Martin Trtilek, Ian Bancroft, Jan Hejatko

**Affiliations:** CEITEC - Central European Institute of Technology, Masaryk University, Kamenice 5/A2, 625 00 Brno, Czech Republic; National Centre for Biomolecular Research, Faculty of Science, Masaryk University, Kamenice 5/A2, 625 00 Brno, Czech Republic; CEITEC - Central European Institute of Technology, Masaryk University, Kamenice 5/A2, 625 00 Brno, Czech Republic; National Centre for Biomolecular Research, Faculty of Science, Masaryk University, Kamenice 5/A2, 625 00 Brno, Czech Republic; Institute of Cytology and Genetics, Siberian Branch, Russian Academy of Sciences, Novosibirsk, 630090, Russia; Faculty of Natural Sciences, Novosibirsk State University, Novosibirsk, 630090, Russia; Institute of Cytology and Genetics, Siberian Branch, Russian Academy of Sciences, Novosibirsk, 630090, Russia; Faculty of Natural Sciences, Novosibirsk State University, Novosibirsk, 630090, Russia; CEITEC - Central European Institute of Technology, Masaryk University, Kamenice 5/A2, 625 00 Brno, Czech Republic; CEITEC - Central European Institute of Technology, Masaryk University, Kamenice 5/A2, 625 00 Brno, Czech Republic; Department of Biology, University of York, York, UK; PSI (Photon Systems Instruments), Ltd, Drásov, 66424 Drásov, Czech Republic; PSI (Photon Systems Instruments), Ltd, Drásov, 66424 Drásov, Czech Republic; Department of Biology, University of York, York, UK; CEITEC - Central European Institute of Technology, Masaryk University, Kamenice 5/A2, 625 00 Brno, Czech Republic; National Centre for Biomolecular Research, Faculty of Science, Masaryk University, Kamenice 5/A2, 625 00 Brno, Czech Republic; University of Warwick, UK

**Keywords:** *Arabidopsis thaliana*, *Brassica napus*, *Brassica oleracea*, *Brassica rapa*, cytokinins, multistep phosphorelay, osmotic stress, salinity, two-component signaling, type-A response regulator

## Abstract

Multistep phosphorelay (MSP) signaling integrates hormonal and environmental signals to control both plant development and adaptive responses. Type-A *RESPONSE REGULATOR* (*RRA*) genes, the downstream members of the MSP cascade and cytokinin primary response genes, are thought to mediate primarily the negative feedback regulation of (cytokinin-induced) MSP signaling. However, transcriptional data also suggest the involvement of *RRA* genes in stress-related responses. By employing evolutionary conservation with the well-characterized *Arabidopsis thaliana RRA* genes, we identified five and 38 novel putative *RRA* genes in *Brassica oleracea* and *Brassica napus*, respectively. Our phylogenetic analysis suggests the existence of gene-specific selective pressure, maintaining the homologs of *ARR3*, *ARR6*, and *ARR16* as singletons during the evolution of *Brassicaceae*. We categorized *RRA* genes based on the kinetics of their cytokinin-mediated up-regulation and observed both similarities and specificities in this type of response across *Brassicaceae* species. Using bioinformatic analysis and experimental data demonstrating the cytokinin and abiotic stress responsiveness of the *A. thaliana*-derived *TCSv2* reporter, we unveil the mechanistic conservation of cytokinin- and stress-mediated up-regulation of *RRA* genes in *B. rapa* and *B. napus*. Notably, we identify partial cytokinin dependency of cold stress-induced *RRA* transcription, thus further demonstrating the role of cytokinin signaling in crop adaptive responses.

## Teaser

We identified *Brassica napus* homologs of *Arabidopsis thaliana* type-A response regulator (*RRA* ) genes, and demonstrated the existence of selective pressure preventing their multiplication during *Brassicaceae* evolution, and the cytokinin dependency of their cold-induced up-regulation.

## Introduction

Cytokinins regulate a wide range of biological processes that are vital for plant growth and development (Werner and Schmulling, 2009; Zurcher and Muller, 2016; Cortleven *et al.*, 2019). In *Arabidopsis thaliana*, cytokinin signaling occurs through a multistep phosphorelay (MSP), sometimes also called two-component signaling (Kieber and Schaller, 2018). The core components of MSP include ARABIDOPSIS HISTIDINE KINASEs (AHKs), ARABIDOPSIS HISTIDINE-CONTAINING PHOSPHOTRANSMITTERs (AHPs), and ARABIDOPSIS RESPONSE REGULATORs (ARRs). In the presence of cytokinins, the CHASE-containing AHKs (AHK2, AHK3, and AHK4) located at the plasma membrane or endoplasmic reticulum (ER) undergo autophosphorylation at a conserved His residue and transfer the phosphate group to the conserved Asp residue within the AHK receiver domain (Hwang and Sheen, 2001; Inoue *et al.*, 2001; Muller and Sheen, 2007; Antoniadi *et al.*, 2020; Kubiasova *et al.*, 2020). Cytoplasmic AHPs accept the phosphate from the AHKs and translocate to the nucleus, allowing the final transphosphorylation of the receiver domain of type-B RRs (RRBs) and transcriptional regulation of the cytokinin-responsive genes.

In addition to the aforementioned RRBs, the *A. thaliana* genome contains two more types of RRs: type-A RRs (RRAs) and type-C RRs (RRCs; Imamura *et al.*, 1998; Schaller *et al.*, 2008). RRBs possess a cytokinin-responsive receiver domain along with a large C-terminal extension that harbors the GARP (Golden/ARR/Psr1) motif, a Myb-like DNA-binding domain (Hosoda *et al.*, 2002). In contrast, the RRAs are characterized by the presence of a receiver domain and short C-terminal sequences but do not contain the DNA-binding domain. *RRA* genes act as cytokinin primary response genes, being rapidly induced by cytokinins via direct transcriptional activation by RRBs, even in the absence of *de novo* protein synthesis (Taniguchi *et al.*, 1998; D’Agostino *et al.*, 2000). RRA proteins are phosphorylated by RRBs and mediate the negative regulation of MSP signaling via as yet unknown mechanisms (Lee *et al.*, 2008). There are 10 known *RRA* genes in *A. thaliana* (*ARR3*, *ARR4*, *ARR5*, *ARR6*, *ARR7*, *ARR8*, *ARR9*, *ARR15*, *ARR16*, and *ARR17*), acting as partially redundant negative regulators of (cytokinin-induced) MSP signaling (To *et al.*, 2004).

Previous studies have demonstrated the key role of *A. thaliana RRA* genes in several developmental and growth regulatory processes including stem cell specification, meristem activity, and regeneration (Leibfried *et al.*, 2005; Muller and Sheen, 2008; Buechel *et al.*, 2010; Zhao *et al.*, 2010). In addition, the transcriptional activity of *RRA* genes was shown to be linked to diverse abiotic stress responses, including salinity, cold, and drought (Urao *et al.*, 1998; Jain *et al.*, 2006; Tran *et al.*, 2007; Jeon *et al.*, 2010; Kang *et al.*, 2012; Shi *et al.*, 2012; Sharan *et al.*, 2017; Wang *et al.*, 2019; Bhaskar *et al.*, 2021). For instance, exposure to cold and dehydration stress triggers the up-regulation of *ARR5*, *ARR6*, *ARR7*, and *ARR15*. These *RRA* genes were shown to play a negative role in cold and dehydration stress regulation in *A. thaliana* (Jeon *et al.*, 2010; Kang *et al.*, 2012). Furthermore, overexpression of the rice *RRA* gene *OsRR6* increased drought and salinity tolerance in *A. thaliana* (Bhaskar *et al.*, 2021). All the aforementioned findings suggest the important role of *RRA* genes in abiotic stress responses. However, the role of cytokinins and/or cytokinin signaling in the regulation of stress-mediated up-regulation of *RRA* genes is not clear.

Advancements in sequencing technologies have facilitated the genome-wide identification of putative components of the MSP cascade not only in *A. thaliana* (Hwang and Sheen, 2001) but also in crop species such as rice (Ito and Kurata, 2006; Jain *et al.*, 2006; Pareek *et al.*, 2006; Karan *et al.*, 2009; Tsai *et al.*, 2012; Sharan *et al.*, 2017), maize (Asakura *et al.*, 2003), soybean (Mochida *et al.*, 2010), and wheat (Sun *et al.*, 2022). Members of the *Brassica* family are among the most commercially valuable species, as both culinary vegetables and oilseed crops, covering ~38 Mha globally (Kumar *et al.*, 2009; European Commission, 2019; Rathore *et al.*, 2022). Several genes involved in MSP signaling have been reported in Chinese cabbage [*B. rapa* spp. Pekinensis (Liu *et al.*, 2014; Kaltenegger *et al.*, 2018)], *B. oleracea* (Kaltenegger *et al.*, 2018), and *B. napus* (Kuderova *et al.*, 2015; Jiang *et al.*, 2022). However, a comprehensive characterization of *RRA* genes and their orthologs across important crop species is lacking within the current scientific literature. Considering the transcriptional activation of *RRA* genes as a dynamic readout of nearly immediate changes in MSP activity (D’Agostino *et al.*, 2000; Hejatko *et al.*, 2009; Pernisova *et al.*, 2009), this represents a substantial gap in our understanding of the role of MSP signaling in the control of plant development and adaptive responses.

In this study, we identify novel *RRA* genes in *B. napus* and *B. oleracea* and provide insights into the evolutionary relationships, kinetics, and mechanism of cytokinin responses, as well as the involvement of cytokinin in the abiotic stress-mediated modulation of *RRA* genes within *A. thaliana* and *Brassica* species.

## Materials and methods

### Identification of type-A response regulators in *Brassica* species, motif search, multiple sequence alignment, and chromosomal mapping

The protein sequences of the 10 known type-A RRs in the *Arabidopsis thaliana* genome (Hwang *et al.*, 2002) were obtained from NCBI (https://www.ncbi.nlm.nih.gov/protein/) (NCBI reference sequence ARR3 NP_176202.1, ARR4 NP_001321924.1, ARR5 NP_190393.1, ARR6 NP_201097.1, ARR7 NP_173339.1, ARR8 NP_181663.1, ARR9 NP_001325622.1, ARR15 NP_177627.1, ARR16 NP_181599.1, and ARR17 NP_567037.1) (Supplementary Table S1). These sequences were used as queries in Protein BLAST (BLASTP) searches against the protein database of *B. oleracea*, *B. rapa*, and *B. napus* in EnsemblPlants (Release 51) (Howe *et al.*, 2021). Genes were selected as described by Kaltenegger *et al.* (2018). The coding sequences, genomic sequences, and protein sequences of the selected genes were retrieved from EnsemblPlants (Release 51) (Howe *et al.*, 2021) and Brassicaceae Database (BRAD version 3.0; http://brassicadb.cn) (Chen *et al.*, 2021).

Using the Expasy SIM-Alignment Tool for protein sequences with BLOSUM62 as a comparison matrix (https://web.expasy.org/sim/) (Duvaud *et al.*, 2021), the amino acid sequence homology of the identified *Brassica* RRAs was compared with *A. thaliana* RRAs (Supplementary Table S2). Similarly, the *B. napus RRA* genes from both A and C subgenomes were compared with those of their progenitor species *B. rapa* and *B. oleracea.* The presence of the conserved response regulator domain was analyzed using the GenomeNet Bioinformatics Tools, sequence motif search, MOTIF (https://www.genome.jp/tools/motif/) of Kyoto University Bioinformatics Center. The protein sequences of the identified *Brassica RRA* genes were used as input, and a search against the PFAM database was performed with a cut-off score of E-value=1. Sequences that possessed the conserved response regulator receiver (Rec) domain (PF00072) were selected for further analysis in this study.

Multiple sequence alignment was conducted using the MUSCLE algorithm (Edgar, 2004) implemented in UGENE (Okonechnikov *et al.*, 2012) to annotate the location of important conserved residues. The genomic locations of *A. thaliana* and *Brassica RRA* genes were retrieved from EnsemblPlants (Release 51) (Howe *et al.*, 2021) and BrassicaDB (BRAD version 3.0; http://brassicadb.cn) databases (Chen *et al.*, 2021). These locations were visualized using MapGene2Chrom (MG2C_v2.1, http://mg2c.iask.in/mg2c_v2.1/) (Chao *et al.*, 2015) by setting appropriate parameters for the figure output. The identified *Brassica RRA* genes were named following the nomenclature proposed by Heyl *et al.* (2013), and the numbers assigned to them correspond to their *A. thaliana* counterparts after performing phylogenetic analysis. In cases where multiple homologs of *ARR4*, *ARR5*, *ARR7*, *ARR8*, *ARR9*, *ARR15*, and *ARR17* were found in *Brassica*, they were designated with the letters ‘a’, ‘b’, or ‘c’ in descending order of homology depending on the percentage amino acid identities they share with that specific RRA.

### Phylogenetic analysis of type-A response regulator genes and gene structure analysis

A comparative phylogenetic analysis was conducted using MEGA7 (Kumar *et al.*, 2016) based on the alignment of the conserved Rec domain (PF00072) as described by Kaltenegger *et al.* (2018). The multiple sequence alignment was performed using the conserved Rec domain employing the MUSCLE algorithm (Edgar, 2004) implemented in MEGA7 (Kumar *et al.*, 2016). The Neighbor–Joining method (Saitou and Nei, 1987) was used to infer the evolutionary history. The evolutionary distances were computed using the Poisson correction method (Zuckerkandl and Pauling, 1965) and are expressed as the number of amino acid substitutions per site. The analysis included 1000 bootstrap replicates, and all ambiguous positions were removed for each sequence pair. Phylogenetic trees were constructed to compare the individual *Brassica* species with *A. thaliana RRA* genes, as well as to compare all the *Brassica RRA* genes among themselves. Gene structure analysis of *A. thaliana* and *Brassica RRA* genes including their schematic representations was made using Gene Structure Display Server (http://gsds.gao-lab.org/) (Hu *et al.*, 2015).

Dual synteny plots were created using the TBTools dual synteny plot function (Chen *et al.*, 2020) to compare the *Brassica* species with *A. thaliana*, and *B. napus* with its parental species, *B. rapa* and *B. oleracea.* Before plotting the dual synteny, a one-step MCScanX analysis was performed in TBTools. The genome sequence files and gene structure annotation files for *Brassica* species and *A. thaliana* were retrieved from EnsemblPlants (Release 54) (Cunningham *et al.*, 2021).

### Plant materials, hormones, and abiotic stress treatment

Seeds of *A. thaliana* (Col-0), *B. rapa* (R-0-18), *B. oleracea* (DH1012), and *B. napus* (Darmor) were cultivated on 1/2 Murashige and Skoog (MS) medium for 1 week inside a growth chamber under controlled conditions. Before cultivation, the seeds underwent a cold pre-treatment in darkness at 4 °C for 3 d. The growth chamber was maintained at a temperature of 21 °C /18 °C for a 16 h day/8 h night photoperiod, with 130 µmol m^–2^ s^–1^ light intensity.

To investigate the expression profile of the 10 *A. thaliana RRA* and 66 *Brassica RRA* genes after cytokinin treatment, 1-week-old seedlings were exposed to exogenous treatment with 5 µM 6-benzylaminopurine (BAP) for 0, 0.5, 1, 2, and 4 as described by D’Agostino *et al.* (2000).

For the abiotic stress treatment, 1-week-old were incubated at 4 °C in the presence of white light for cold treatment. For salinity stress, the seedlings were treated with a 250 mM NaCl solution, and for osmotic stress, the seedlings were treated with a 300 mM mannitol solution. For the control treatment, the seedlings were treated with water only. Both the control and stress-treated seedlings were incubated in the growth chamber with a set temperature of 21 °C, with a light intensity of 130 µmol m^–2^ s^–1^ for 2 h and 4 h.

Additionally, a separate cold treatment experiment was conducted following the methodology described above to assess the expression of cold-responsive *ARR7* and its *Brassica* homologs. The focus of this experiment was to evaluate the effects of the purine derivative PI-55, a known antagonist of cytokinin receptor activity (Spichal *et al.*, 2009). One-week-old seedlings were treated with either PI-55 (0.1 µM and 1 µM) or 0.1% DMSO, and incubated under either cold (4 °C) or control conditions (21 °C) for 4 h.

### RNA isolation and quantitative reverse transcription–PCR analysis

Total RNA was extracted from the collected seedlings following the Quick-Start Protocol included in the RNeasy® Plant Mini Kit (QIAGEN, Germany). Additionally, DNase treatment was performed using an RNase-Free DNase set (QIAGEN) to remove any DNA contamination. The concentration, integrity, and purity of the extracted RNA samples were examined using a NanoDrop One UV spectrophotometer (Thermo Fisher Scientific). Reverse transcription was performed to generate first-strand cDNA using the SuperScript™ III First-Strand Synthesis System (Thermo Fisher Scientific) with 1 µg of RNA using oligo(dT) primer. For the expression profiling of *RRA* genes after cytokinin treatment and abiotic stress exposure, 66 out of the 78 *Brassica RRA* genes along with the 10 *A. thaliana RRA* genes were analyzed. For the expression profiling of cold-responsive *ARR* genes after PI-55 treatment, *ARR7* and its *Brassica* homologs (i.e. *BrRRA7b*, *BoRRA7a*, *BoRRA7b*, *BnARRA7a*, *BnARRA7b*, *BnCRRA7a*, and *BnCRRA7b*) were analyzed. Several reference genes were utilized as an internal control, including commonly used housekeeping genes (Guénin *et al.*, 2009) such as *UBQ10* and *UBC10* (added for abiotic stress) for Arabidopsis, *BrELF1* for *B. rapa*, *BoTUB6* for *B. oleracea*, and *BnACT2A* and *BnACT2C* for *B. napus* (primers listed in Supplementary Table S3). All primers used were designed based on the following features: product size (70–200 bp), primer length (18–22 bp), *T*_m_ (59–65 °C), GC content (50–60%), target gene specificity, and absence of nucleotide repeats. Quantitative reverse transcription–PCRs were performed using FastStart SYBR® Green Master (Roche Diagnostics GmbH) on the Rotor-Gene Q 5plex HRM Platform (QIAGEN, Germany). Melting curve analysis was performed to confirm the specificity of the product for each primer pair. The relative gene expression level was calculated relative to the control using the delta-delta Ct method (Pfaffl, 2004). The RT–qPCR analysis was performed in three independent biological replicates, each with three technical replicates. Subsequently, a heatmap representation of the expression of *RRA* genes after exogenous cytokinin treatment and abiotic stress treatment was generated and is presented as the log2 fold change (log_2_FC). The heatmap was constructed using Cluster 3.0 for Windows (de Hoon *et al.*, 2004) and viewed using Java TreeView (Saldanha, 2004).

### Analysis of *cis*-regulatory elements in the promoter regions of *RRA* genes across *Brassica* species

Multiple sequence alignment of the homologous RRB amino acid sequences from *Brassica* species and *A. thaliana* was performed using Clustal Omega (Madeira *et al.*, 2022) to assess the conservation of their GARP-like DNA-binding domains. The alignment was visualized using the MView online tool (Madeira *et al.*, 2022). Reference genomes and genome annotations for *A. thaliana*, *B. rapa*, *B. oleracea*, and *B. napus* were downloaded from EnsemblPlants (Yates *et al.*, 2022). The upstream regulatory sequences of protein-coding genes were extracted from the reference genomes using GFF3 annotations with the Bedtools getfasta tool (Quinlan and Hall, 2010). The publicly available ChIP-seq data for *A. thaliana* transcription factors (TFs) ARR1 and ARR10 (Xie *et al.*, 2018) was used for a *de novo* motif search with Homer (Heinz *et al.*, 2010). To identify potential RRB-binding sites in gene regulatory regions, Position Weight Matrices (PWMs) were used. The thresholds for PWMs were calculated using the previously described algorithm (Touzet and Varré, 2007). Then the PWMs were applied to three 500 bp long intervals of protein-coding genes: [–1500; –1000], [–1000; –500], and [–500; +1] relative to the transcription start site. To compare the density of potential RRB-binding sites in the regulatory regions of *Brassica* RRA-coding genes (used in the cytokinin and abiotic stress treatment) with random expectation (which is the density of the binding sites in the regulatory regions of all protein-coding genes), Fisher’s exact test was used. To account for multiple testing, we used Bonferroni correction: the *P*-value threshold was set as 0.05/24. The fold enrichment was calculated as the ratio of RRB-binding site density in RRA regulatory regions to the average density in the corresponding regions of all protein-coding genes.

The promoter sequences of *A. thaliana* and *Brassica RRA* genes (used in the cytokinin and abiotic stress treatment) were also subjected to *in silico* analysis using the online database, PlantCARE (http://bioinformatics.psb.ugent.be/webtools/plantcare/html/) (Lescot *et al.*, 2002). The objective was to investigate the presence of environmental stress-responsive *cis*-elements in these sequences. Additionally, a Pearson correlation analysis was conducted to determine the relationship between the gene expression of cold-responsive *A. thaliana RRA* genes (*ARR6*, *ARR7*, and *ARR15*) and *Brassica RRA* genes (*BrRRA6*, *BrRRA7a*, *BrRRA7b*, *BrRRA15a*, *BrRRA15b*, *BoRRA6*, *BoRRA7a*, *BoRRA7b*, *BoRRA15a*, *BoRRA15b*, *BnARRA6*, *BnARRA7a*, *BnARRA7b*, *BnARRA15a*, *BnCRRA6*, *BnCRRA7a*, *BnCRRA7b*, *BnCRRA15a*, and *BnCRRA15b*) after 2 h and 4 h of cold exposure, and the total number of environmental stress-related *cis*-elements within the promoter regions of these genes. In the case of *A. thaliana*, additional comparisons were made using DAPseq data to select TFs with potential binding sites in the *A. thaliana* promoters. Moreover, to assess the enrichment of the TF-binding sites, particularly the PWM models in *A. thaliana*, a comparison was made between stress-sensitive promoters and stress-insensitive promoters for both *A. thaliana* and *Brassica* species.

### Transformation of *Brassica* species with *TCSv2:3×VENUS*, cytokinin, and abiotic stress treatment

The *TCSv2*:*3×VENUS* construct, obtained from Maya Barr (Steiner *et al.*, 2020), was subcloned into the pGREEN00279 binary vector (Hellens *et al.*, 2000) and introduced into *B. rapa* (R-0-18), *B. oleracea* (DH1012), and *B. napus* (Darmor), following the protocol described by Jedlickova *et al.* (2022). Only root tips of *B. rapa* and *B. napus* transformed hairy roots were used in the experiment, as the transformation for *B. oleracea* was unsuccessful. Root tips of *B. rapa* and *B. napus* hairy roots were gathered 2 weeks after subculturing and treated with either 5 µM synthetic BAP or 0.1% DMSO for 0, 0.5, 1, 2, and 4 h, as described by D’Agostino *et al.* (2000), to test the cytokinin responsiveness of *TCSv2*:*3×VENUS* in the *Brassica* species. A total of three biological replicates were performed, with five roots for each replicate.

For stress treatments, the root tips of transformed *B. rapa* and *B. napus* hairy roots were exposed to 4 °C in the presence of white light for cold treatment. For salinity stress, the hairy roots were treated with 250 mM NaCl solution, and for osmotic stress they were treated with a 300 mM mannitol solution. For the control treatment, the hairy roots were treated with water only. Both the control and stress-treated hairy roots were incubated in the growth chamber with a set temperature of 21 °C, with a light intensity of 130 µmol^–2^ s^–1^ for 2 h and 4 h. A total of three biological replicates were performed, with 15 roots per replica.

### Root imaging, and quantification of reporter gene expression

Root tips were imaged using the laser scanning confocal imaging microscope Zeiss LSM780 Axio-Observer, equipped with an external In Tune laser (488–649 nm, <3 nm width, pulsed at 40 MHz, 1.5 mW C-Apochromat) and a ×20 objective. The expression of VENUS in the root apical meristem (RAM) was quantified using IMAGEJ software (Schneider *et al.*, 2012) and the spot detection algorithm in IMARIS 9.0 (Bitplane, http://www.bitplane.com/imaris/imaris). Representative images generated using IMARIS are presented. To ensure accurate analysis, the fluorescence intensity of each DMSO- or BAP-treated root was initially normalized to the area of the scanned roots (in pixels) and further normalized to the fluorescence intensity of the roots at the start of the treatment (0 h). Subsequently, the relative fluorescence intensity was calculated as the ratio of normalized fluorescence intensity in BAP-treated roots to the normalized fluorescence intensity of DMSO-treated roots.

### Statistical analysis

A one-way ANOVA followed by Dunnett’s test was conducted to evaluate differences in the calculated relative fluorescence intensity in the scanned roots at the start and after 0.5, 1, 2, and 4 h of exogenous BAP treatment. Furthermore, a two-way ANOVA followed by Tukey’s HSD multiple comparison test was employed to compare the relative expression of cold-responsive *ARR7*, *BrRRA7a*, *BrRRA7b*, *BoRRA7a*, *BoRRA7b*, *BnARRA7a*, *BnARRA7b*, *BnCRRA7a*, and *BnCRRA7b* after PI-55 treatment. Statistical analysis was conducted using the GraphPad Prism version 9.0 for Windows (GraphPad Software, San Diego, CA, USA).

## Results

### The type-A response regulators and their genomic distribution in the *Brassicaceae* family

**Fig. 1.**
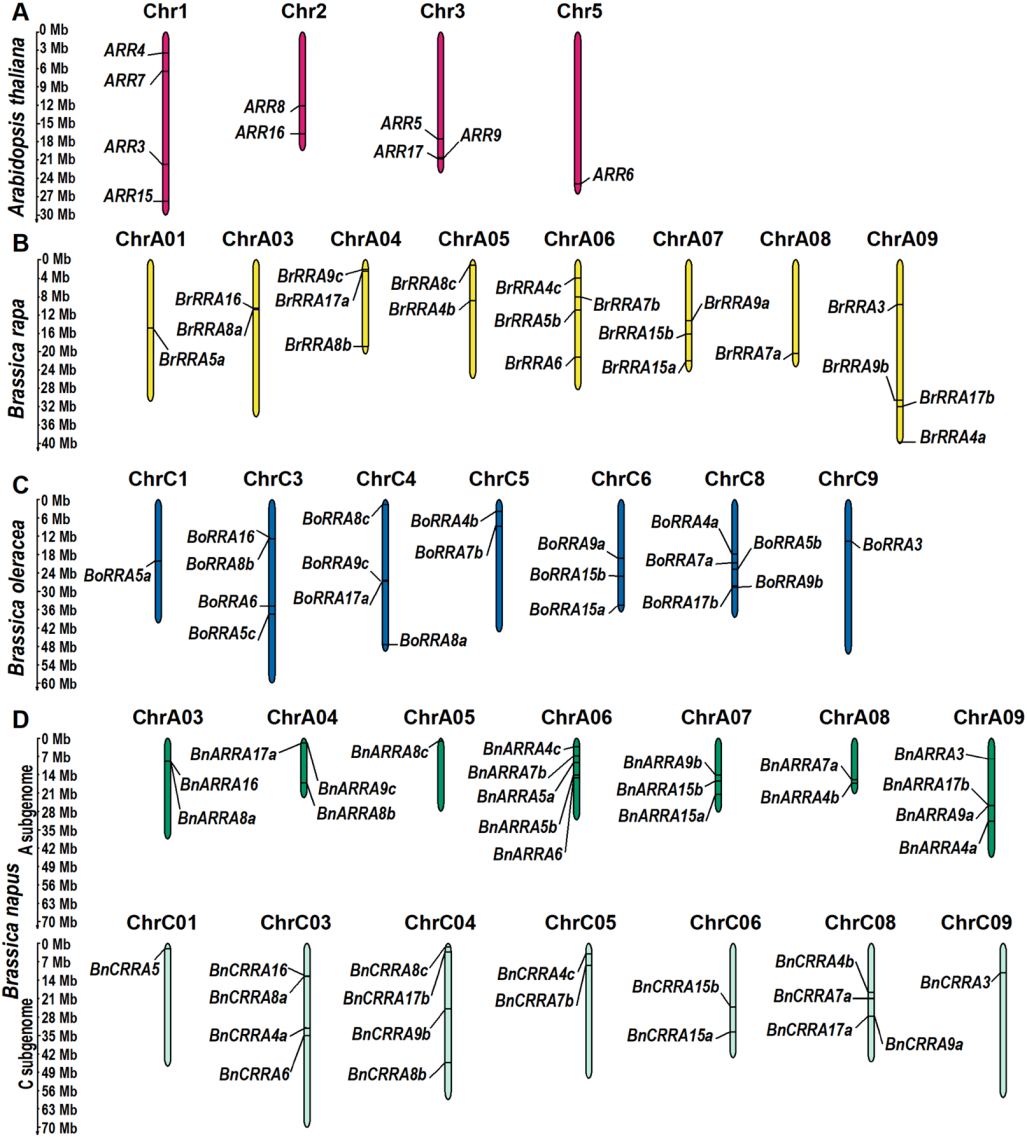
Chromosomal localization of known and newly identified *RRA* genes in *Brassicaceae*. *RRA* genes in (A) *Arabidopsis thaliana*, (B) *Brassica rapa*, (C) *Brassica oleracea*, and (D) A and C subgenome of *Brassica napus*. Each panel displays only the chromosomes (designated as ‘Chr’) where the *RRA* genes were identified.

Using a similarity search (see the Materials and methods for more details), we identified 78 putative *RRA* genes in *B. oleracea*, *B. rapa*, and *B. napus* that share a high degree of sequence identity with *A. thaliana RRA* genes (Fig. 1A–D; Supplementary Table S2). Among these, 20 and 15 were previously reported in the genome of *B. rapa* and *B. oleracea*, respectively (Liu *et al.*, 2014; Kaltenegger *et al.*, 2018), thus affirming the robustness of our bioinformatic search methodology. Following previously agreed nomenclature (Heyl *et al.*, 2013), we designated them as *BrRRA* and *BoRRA* genes (Fig. 1B, C). In the genome of *B. oleracea*, we found five novel putative *RRA* genes that were not included in Kaltenegger *et al.* (2018) (Fig. 1C). In *B. napus*, we recognized 38 novel putative *RRA* genes, 20 of which located in the A subgenome (*BnARRA* genes) and 18 in the C subgenome (*BnCRRA* genes) (Fig. 1D). The putative paralogs were indexed with ‘a’, ‘b’, or ‘c’ in an order following the decreasing percentage of amino acid identities they share with the corresponding *A. thaliana* RRA (ARR; Fig. 1A).

*BrRRA* genes were mapped to chromosomes ChrA01, ChrA03, ChrA04, ChrA05, ChrA06, ChrA07, ChrA08, and ChrA09, while *BoRRA* genes were located on ChrC1, ChrC3, ChrC4, ChrC5, ChrC6, ChrC8, and ChrC9 (Fig. 1B, C). As expected, *BnARRA* and *BnCRRA* genes were found on corresponding homologous chromosomes in the A and C subgenomes, respectively (ChrA03, ChrA04, ChrA05, ChrA06, ChrA07, ChrA08, and ChrA09 for *BnARRA* genes, and ChrC01, ChrC03, ChrC04, ChrC05, ChrC06, ChrC08, and ChrC09 for *BnCRRA* genes; Fig. 1D).

### *Brassica* and *A. thaliana* RRAs show a high level of conservation

**Fig. 2.**
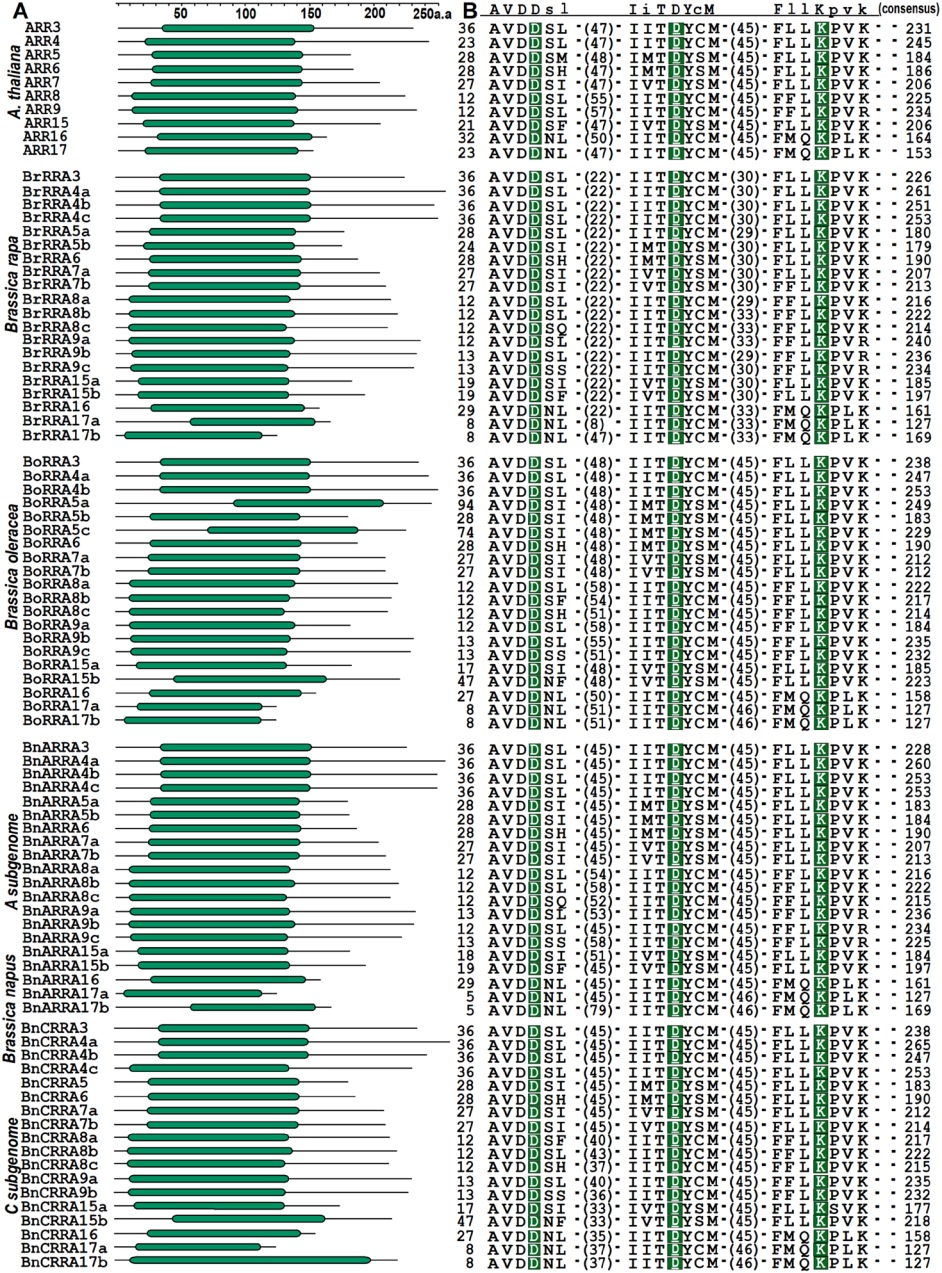
*Arabidopsis thaliana* and *Brassica RRA* genes reveal a high level of domain structure and amino acid sequence conservation. (A) Schematic depiction of the protein domain of RRAs from *A. thaliana* and *Brassica* sp., showing the localization of the receiver domain (Rec, as a green rounded rectangle, and the rest of the amino acid residue as lines). The top line shows the amino acid residue (a.a) position coordinates. (B) Multiple sequence alignment of several amino acid sequences adjacent to the conserved D-D-K motif (green box) in the Rec domain of the individual RRA protein sequences. The numbers of amino acid residues preceding the residues shown in the figure (numbers at the start and in the middle of RRA protein sequences), along with the total number of amino acid residues for each RRA protein sequence (number at the end), are indicated. The consensus sequence is displayed above the alignment; conserved residues are in uppercase, while lowercase characters represent the most common amino acids at variable positions.

**Fig. 3.**
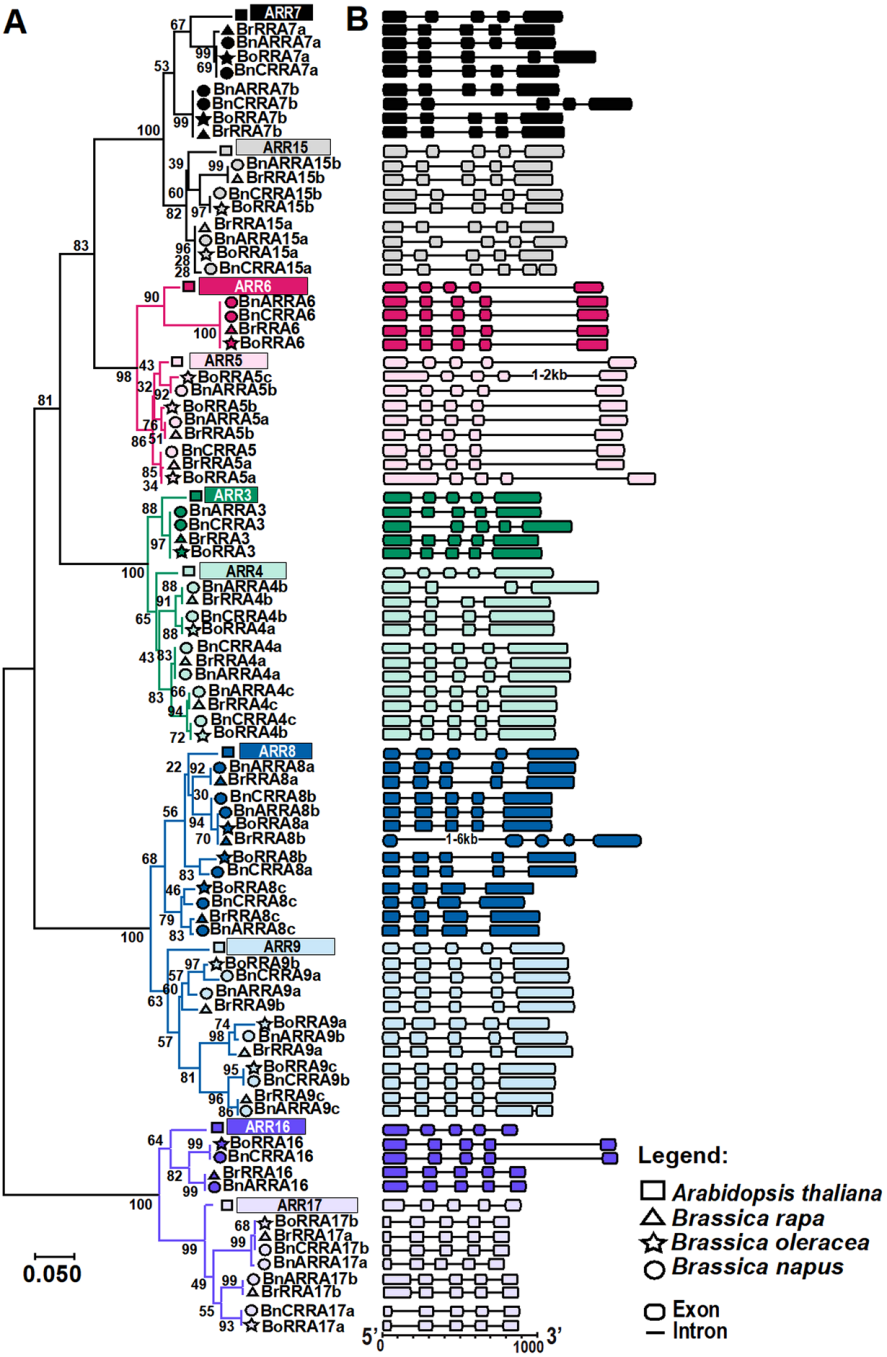
Phylogenetic relationships and gene structures of *RRA* genes in *Brassicaceae*. (A) The unrooted tree is based on the similarity of RRA Rec domains constructed using the Neighbor–Joining method; the bar represents the relative divergence of the examined sequences. The subclades composed of RRAs potentially orthologous to individual *A. thaliana RRA* genes are presented using the same color; the subclades comprising homologs of the paired *A. thaliana RRA* genes, the result of an α WGD event (see the main text for details), are distinguished by different shades of a given color. The RRAs from individual species are distinguished by a triangle (BrRRAs), star (BoRRAS), and circle (BnRRAs). (B) A schematic representation of the *A. thaliana* and *Brassica RRA* gene structures (exons are depicted as boxes separated by introns as lines); the color code is used as in (A).

A motif search in the putative protein sequences of all the 78 *Brassica* RRAs confirmed the presence of the conserved Rec domain harboring the highly conserved D-D-K motif, including the (underlined) phosphoaccepting Asp, which is essential for the role of RRAs in mediating the negative feedback regulation of cytokinin signaling (Lee *et al.*, 2008) (Fig. 2A, B). Moreover, all the predicted *Brassica* RRAs had protein sizes comparable with their putative *A. thaliana* orthologs (identified based on their phylogenetic analysis, see later in the text and Fig. 3), ranging from 127 to 265 amino acid residues, with ARR4 and ARR17 and their homologs being the longest and shortest, respectively (Fig. 2; Supplementary Table S2). The evolutionary relationship among the RRAs (78 *Brassica* and 10 *A. thaliana* RRAs) was assayed by aligning the amino acid sequences of conserved Rec domains (Fig. 3A; Supplementary Figs S1–S3). As expected, we observed a high level of conservation between the RRAs from *Brassica* sp. and *A. thaliana*. The tree consists of five main clades, each composed of two subclades, reflecting the presence of five couples of very similar/paralogous RRAs (ARR7/ARR15, ARR5/ARR6, ARR3/ARR4, ARR16/ARR17, and ARR8/ARR9). This information was used to designate the individual *Brassica* RRAs according to their clustering into individual paralogous subclades (Fig. 3A).

**Fig. 4.**
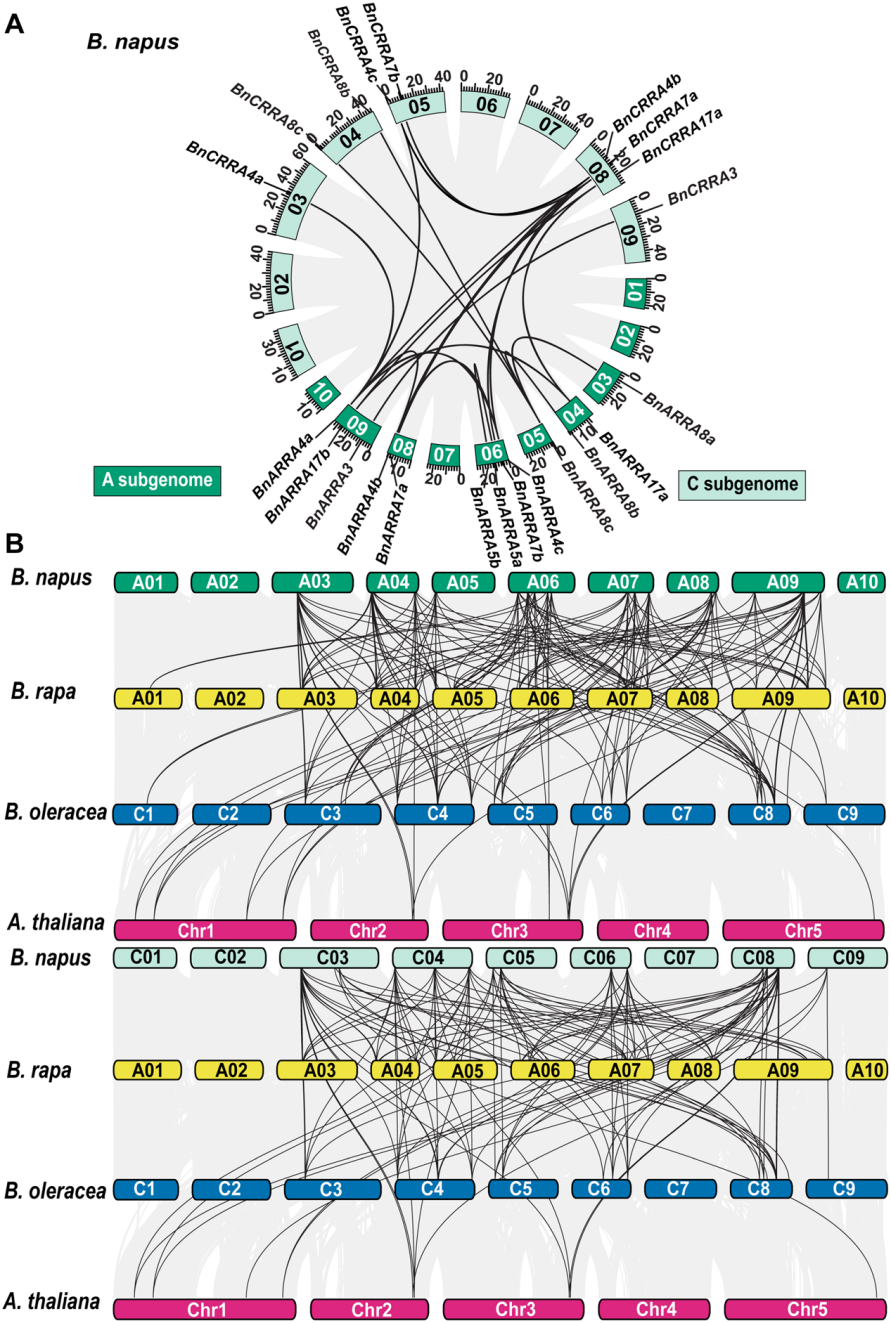
The syntenic conservation of *B. napus RRA* genes. (A) Synteny of the *BnRRA* genes. Gray lines represent syntenic blocks in the *B. napus* genome, while black lines indicate paralogous *BnRRA* gene pairs, demonstrating segmental duplication between different chromosomes. The A and C subgenomes are distinguished by the color difference in the box bearing the chromosome name. The scale at the bottom of these boxes represents the size of the chromosome in megabases. (B) Collinearity of *B. napus* (A and C subgenome), *B. rapa*, *B. oleracea*, and *A. thaliana* genomes. Gray lines illustrate collinear blocks among these species, while black lines show the orthology in the *BnRRA*, *BrRRA*, *BoRRA*, and *A. thaliana RR* genes. The dark and light green boxes represent the chromosomes in the A and C subgenomes of *B. napus*, the yellow boxes for the *B. rapa* chromosomes, the blue boxes for the *B. oleracea* chromosomes, and the dark pink boxes for the *A. thaliana* chromosomes (designated as ‘Chr’).

The analysis of gene structure revealed that, except for eight *RRA* genes containing only four exons (*BrRRA4b*, *BoRRA4a*, *BnARRA4b*, *BnCRRA4b*, *BoRRA8c*, *BrRRA8c*, *BnCRRA8c*, and *BnARRA8c*), all other *RRA* genes shared a gene model consisting of five exons and four introns (Fig. 3B). Among these, *ARR6* and its *Brassica* homologs exhibited nearly identical gene structures, including the number and length of exons and introns. Furthermore, genome-to-genome synteny analysis between the individual *Brassica* species and *A. thaliana* (Fig. 4A) revealed that 20 out of 20 *BrRRA* genes, 11 out of 20 *BoRRA* genes, and 32 out of 38 *BnRRA* genes genes were syntenic with their *A. thaliana* counterparts. In the case of *B. napus*, 36 out of 38 *BnRRA* genes were syntenic with those of *B. rapa* and *B. oleracea*. Within *B. napus* subgenomes, 18 paralogous gene pairs displayed segmental duplications. Among these, nine pairs were segmental duplications between the six *BnARRA* and seven *BnCRRA* genes, two pairs were segmental duplications involving four *BnCRRA* genes, and seven pairs were segmental duplications between 10 *BnARRA* genes (Fig. 4A).

Taken together, a high level of amino acid sequence conservation was observed within the *Brassica* species, confirming the previously described evolutionary relationships (Morinaga, 1929; Nagaharu and Nagaharu, 1935; Cheng *et al.*, 2012, 2014; Nikolov *et al.*, 2019; Hendriks *et al.*, 2023).

### Cytokinin treatment revealed the shared and distinct patterns of the *RRA* expression profiles between *A. thaliana* and *Brassica* sp.

**Fig. 5.**
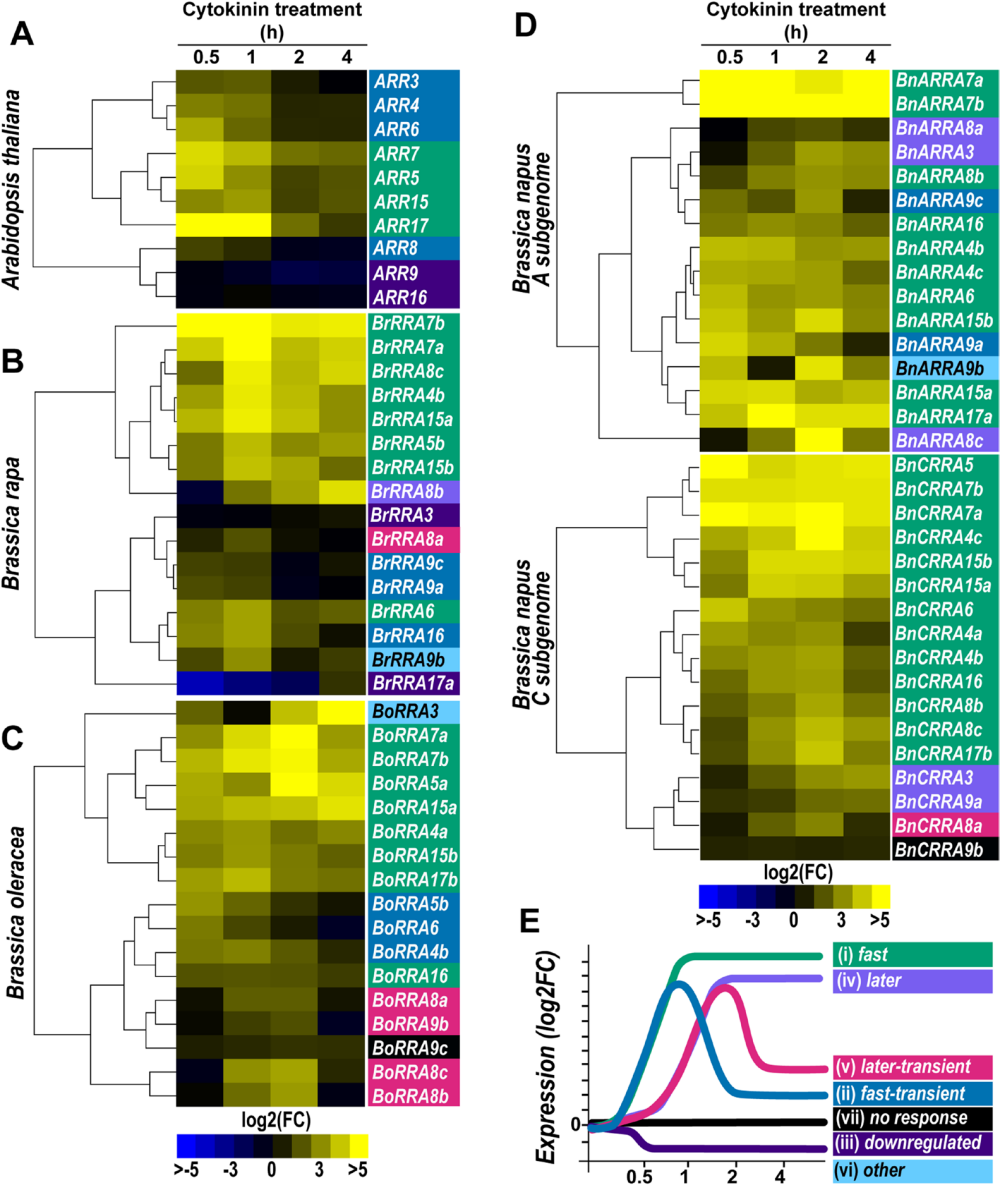
Kinetics of *A. thaliana* and *Brassica RRA* gene response to cytokinins. Heatmaps represent the relative change of *RRA* expression in the 1-week-old seedlings after cytokinin (5 µM BAP) treatment for the given time (0.5, 1, 2, and 4 h) normalized to mock-treated controls in (A) *A. thaliana*, (B) *B. rapa*, (C) *B. oleracea*, and (D) *B. napus*. The expression data are presented as log2 fold change between BAP- and mock-treated samples normalized by the delta-delta Ct (Pfaffl, 2004). (E) Schematic depiction of identified expression profile categories. The categorization of individual RRAs in (A–D) is color-coded as defined in (E).

The *A. thaliana RRA* genes are considered primary cytokinin response genes, as their transcription is promptly induced by exogenous cytokinins even in the absence of *de novo* protein synthesis (Taniguchi *et al.*, 1998; D’Agostino *et al.*, 2000). To compare the effects of cytokinin on the expression of *A. thaliana* and *Brassica RRA* genes, 1-week-old *A. thaliana* and *Brassica* seedlings were exposed to exogenous cytokinins for various times ranging from 30 min to 4 h (Fig. 5; Supplementary Table S4).

Based on the time course of the observed transcriptional response, the expression profiles of individual *A. thaliana RRA* genes were classified into three categories: (i) fast, exhibiting prompt up-regulation after 30 min of cytokinin treatment followed by a gradual decline of expression throughout the rest of the treatment period; (ii) fast-transient, similar to (i), but revealing a fast decline after the initial peak; and (iii) down-regulated, indicating a reduced expression throughout the experiment (Fig. 5A, E; Supplementary Table S4). In *A. thaliana*, we observed the same number (four) of *RRA* genes with cytokinin response profiles classified as fast and fast-transient and two *RRA* genes belonging to the down-regulated category (Fig. 5A). In contrast, in *B. rapa* and *B. oleracea*, the proportion of *RRA* genes with the fast profile increased at the expense of the fast-transient. Additionally, four additional categories emerged: (iv) later, characterized by delayed up-regulation occurring after 1 h of cytokinin treatment and persisting until 4 h; (v) later-transient, similar to the later category but with a decline in expression at 4 h; (vi) other, showing various response types; and (vii) no response (Fig. 5B–D). The decrease in the number of *RRA* genes of the fast-transient category was more pronounced in *B. oleracea* compared with *B. rapa.* This trend was even more evident when comparing the A and C subgenome-specific *RRA* genes in *B. napus*, where at least two *RRA* genes of the fast-transient profile were still retained among the *BnARRA* genes (encoded by the A subgenome of *B. rapa* origin), but no fast-transient *RRA* profile was found among *BnCRRA* genes (located in the C subgenome originating from *B. oleracea*; compare Fig. 5B–D).

Analyzing the cytokinin response of individual *RRA* genes across the *Brassica* species and *A. thaliana*, similar expression profiles were observed for *ARR5*, *ARR7*, and *ARR15*, and most of their homologs in *B. rapa*, *B. oleracea*, and *B. napus*. However, a higher level of expression change (log_2_FC) of these *RRA* genes was observed in the *Brassica* species compared with *A. thaliana*, and this trend was apparent in particular for *B. napus* homologs of *ARR7* (Fig. 5; Supplementary Table S4). This aligns with RNA-sequencing profiling results of *B. napus* cultivars using the Renewable Industrial Products from Rapeseed (RIPR) diversity panel (Havlickova *et al.*, 2018), which identified *ARR7* orthologs as one of the most abundant *RRA* genes among the *B. napus* cultivars (Supplementary Fig. S4).

To sum up, all assayed *RRA* genes across the *Brassicaceae* family were up-regulated by cytokinins, demonstrating partially overlapping, but also species-specific temporal expression patterns.

### Cytokinin-induced up-regulation of *Brassica RRA* genes via motifs recognized by RRBs is conserved in *Brassicaceae*

**Fig. 6.**
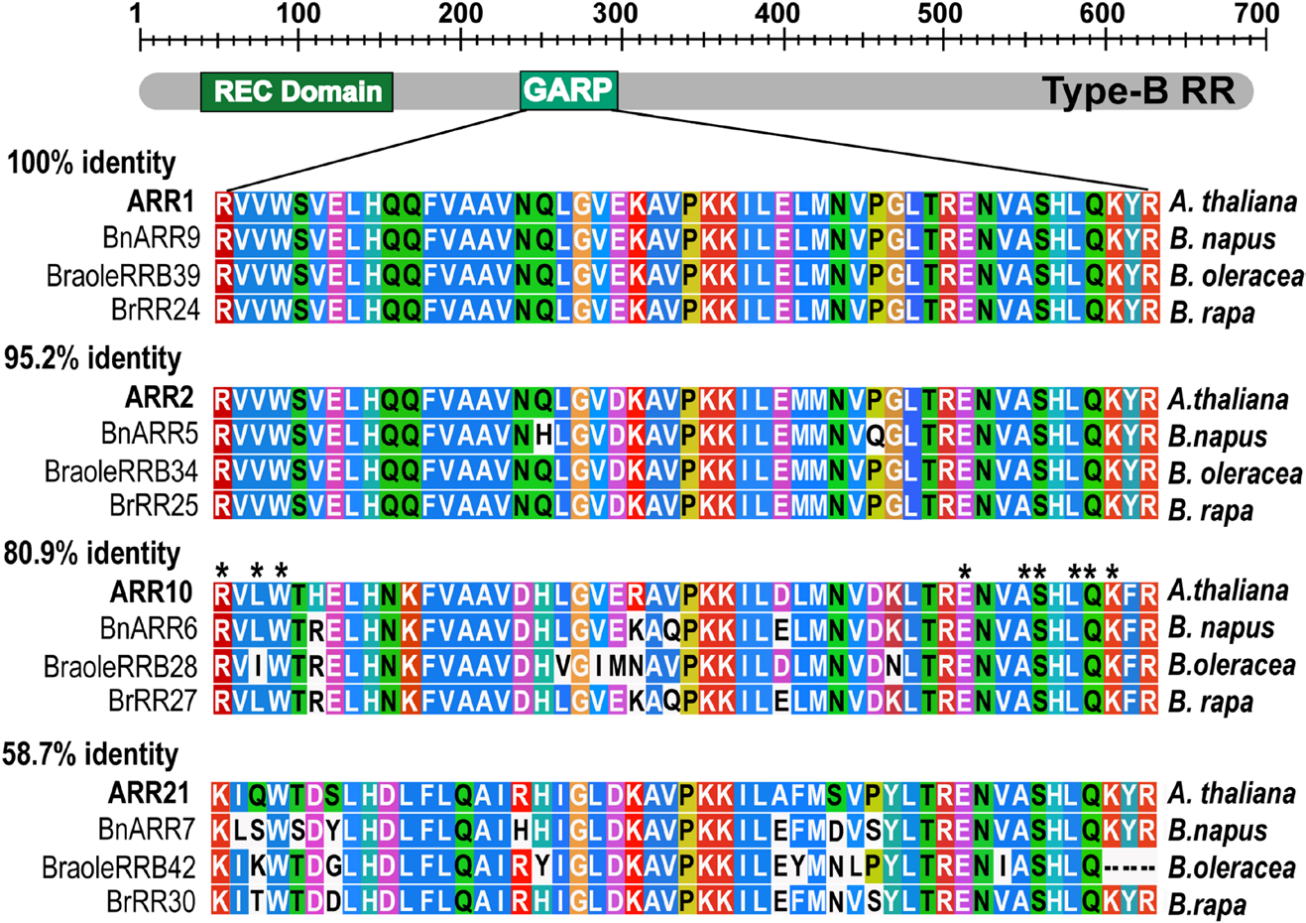
The DNA-binding domain of *A. thaliana* and *Brassica* RRBs shows a high level of amino acid conservation. Domain structure of *A. thaliana* and *Brassica* RRBs and alignment of the amino acid sequences of the GARP-like DNA-binding domain for the selected RRBs from *A. thaliana* and assayed *Brassica* species. Conserved amino acids are highlighted, and the percentage identity is shown. The CLUSTAL color scheme was used to color the alignment, reflecting the physicochemical properties of amino acids (Kunzmann *et al.*, 2020). The asterisk denotes the ARR10 residues proposed to interact directly with DNA (Hosoda *et al.*, 2002); for a comprehensive list of RRB alignments, refer to Supplementary Fig. S5.

In Arabidopsis, cytokinin-dependent transcriptional activation of *RRA* genes is mediated by RRBs, the cytokinin-regulated TFs that bind specific *cis*-regulatory motifs enriched in the promoters of cytokinin-responsive genes (Muller and Sheen, 2008). To assess the possible conservation of DNA targets recognized by RRBs in *A. thaliana* and *Brassica* species, we performed a multiple protein sequence alignment of DNA-binding GARP-like domain of *A. thaliana* RRBs ARR1, ARR2, ARR10, ARR11, ARR12, ARR13, ARR18, ARR19, ARR20, and ARR21 (Sakai *et al.*, 2000; Lohrmann *et al.*, 2001; Hosoda *et al.*, 2002; Mason *et al.*, 2005) and their putative orthologs previously identified in the *Brassica* sp. (Liu *et al.*, 2014; Kaltenegger *et al.*, 2018; Jiang *et al.*, 2022). A high level of conservation was observed, with the identity in amino acid sequence ranging from 100% for ARR1, 95.2% for ARR2, and 80.9% for ARR10, to 58.7% in the case of ARR21 (Fig. 6; Supplementary Fig. S5). Given this high conservation of the GARP-like DNA-binding domain across the *A. thaliana* and *Brassica* RRBs, it is likely that the *Brassica* RRBs recognize DNA-binding motifs similar to those previously described in *A. thaliana* (Sakai *et al.*, 2000; Hosoda *et al.*, 2002; Imamura *et al.*, 2003; Zubo *et al.*, 2017; Xie *et al.*, 2018).

**Fig. 7.**
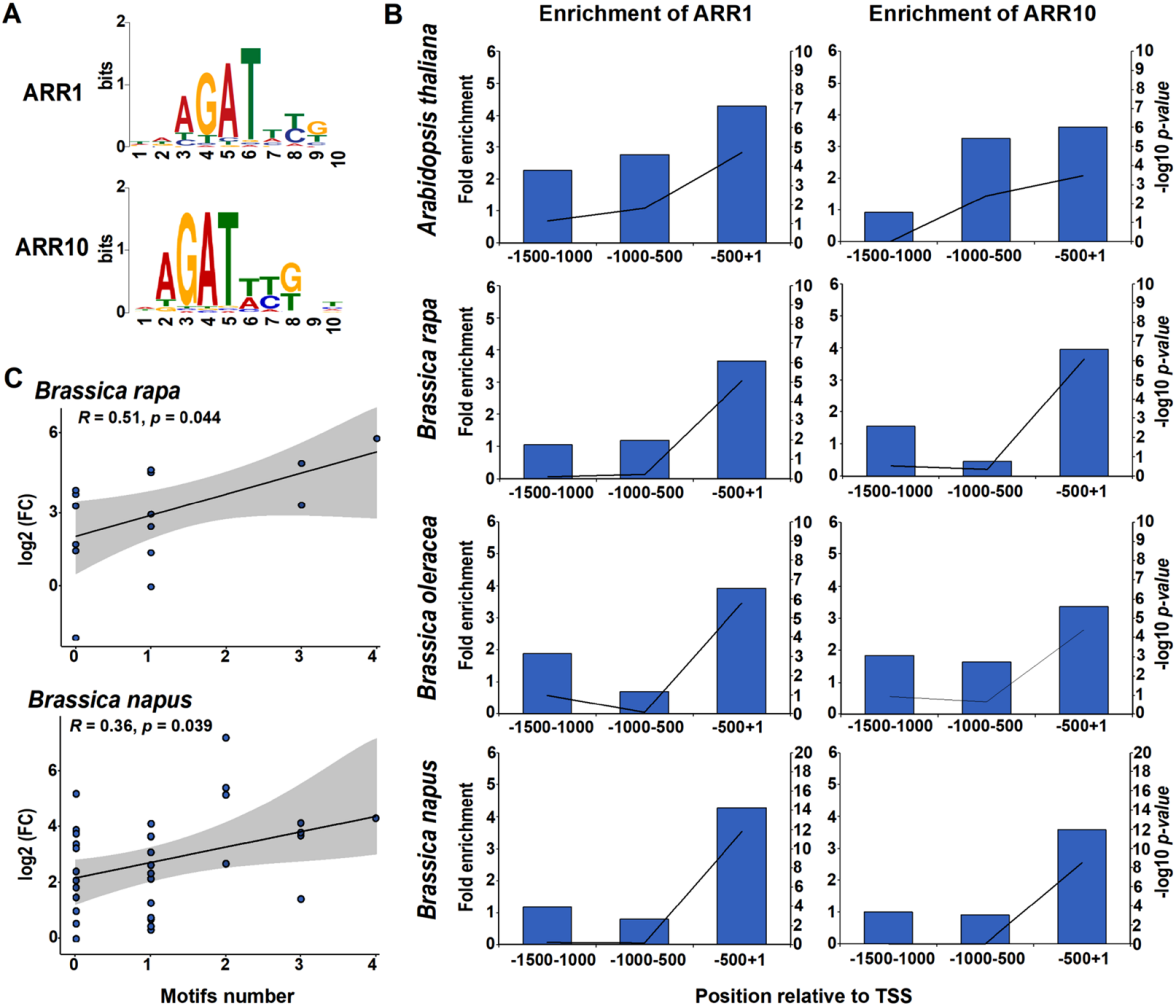
Promoters of *Brassica RRA* genes are enriched for the Arabidopsis-like cytokinin-responsive *cis*-regulatory elements. (A) The Position Weight Matrix (PWM) for the ARR1 and ARR10 DNA-binding sites in *A. thaliana* was retrieved from ChIP-seq peak sets (Xie *et al.*, 2018). (B) Significant enrichment of ARR1 and ARR10 PWM hits proximal to 5'-regulatory regions of *A. thaliana* and *Brassica RRA* genes. Bars represent fold enrichment (left axis) and the line represents –log_10_  *P*-value (right axis). (C) Significant correlation (Pearson correlation with 95% confidence intervals, shadowed part) between the transcriptional response to cytokinin of *BrRRA* and *BnRRA* genes and the number of cytokinin-responsive motifs present in their promoter regions.

To further corroborate this assumption, we utilized the PWMs for the *A. thaliana* ARR1 and ARR10 DNA-binding sites, retrieved from the ChIP-seq peak sets (Zubo *et al.*, 2017; Xie *et al.*, 2018) to predict putative RRB-binding sites within the *Brassica* species (Fig. 7A). Using this approach, the presence of Arabidopsis-like cytokinin-responsive *cis*-elements was predicted in the [–1500; +1 relative to the transcription start site] regulatory regions of 62 out of the 66 analyzed *Brassica RRA* genes used in the cytokinin treatment (Supplementary Table S5). Similar to *A. thaliana*, these potential *cis*-elements were significantly enriched within the proximal 5'-regulatory regions of *Brassica RRA* genes (within 500 bp upstream of the transcription start site; Fig. 7B). We also observed a moderate correlation between the number of motifs within the [–500; +1] regulatory regions and the magnitude of the transcriptional response to cytokinin, which was statistically significant in *B. napus* and *B. rapa* (Fig. 7C). This finding further supports the notion of the functional role of Arabidopsis-like *cis*-elements in regulating the transcriptional response to cytokinins in the assayed *Brassica* species and suggests a possible role for motif clustering in the response amplification.

**Fig. 8.**
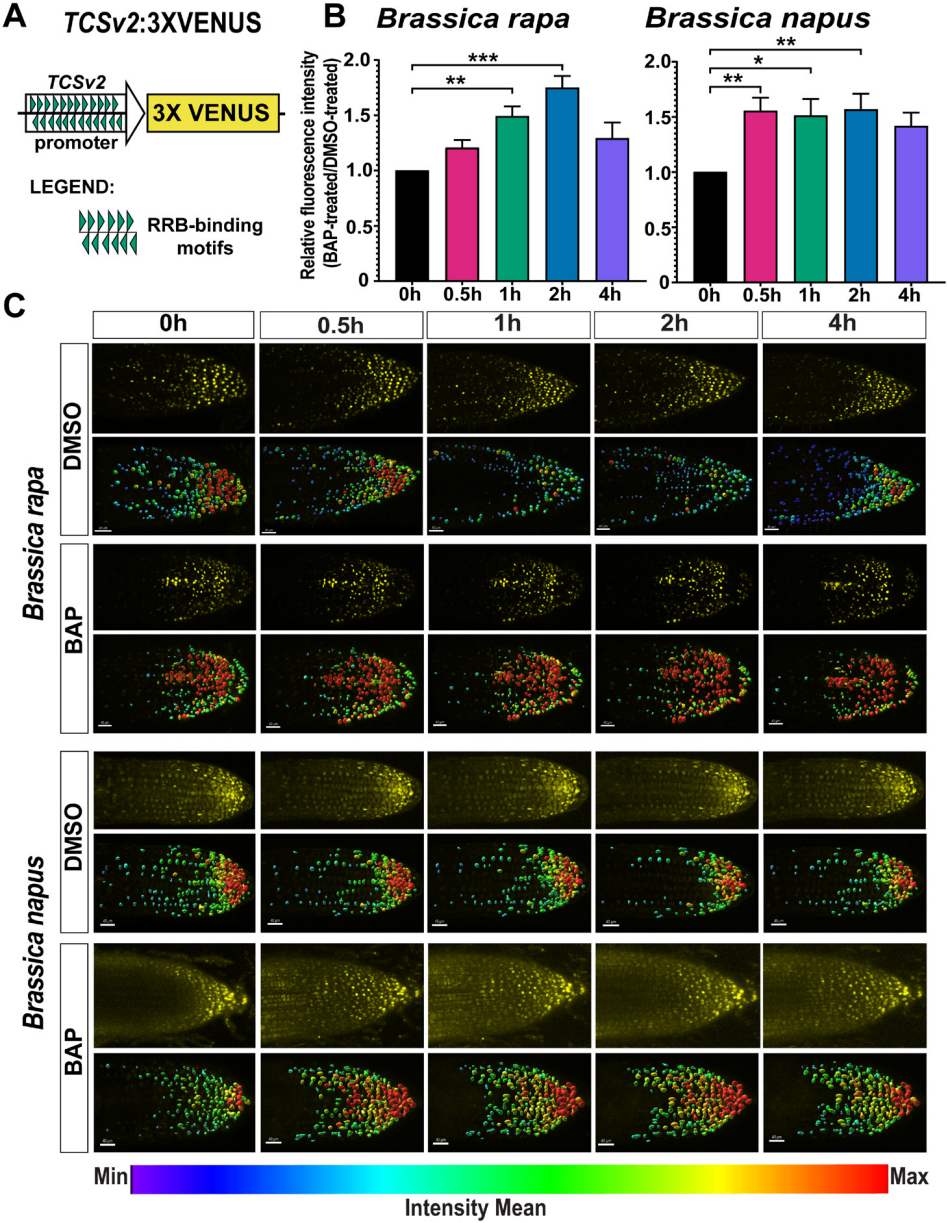
The Arabidopsis *TCSv2*:*3×VENUS* cytokinin reporter (Steiner *et al.*, 2020) is cytokinin responsive in *B. rapa* and *B. napus*. (A) Scheme of the *TCSv2:3×VENUS* (after Steiner *et al.*, 2020). (B) Comparison of the relative fluorescence intensity of the *TCSv2:3×VENUS* cytokinin reporter in BAP-treated hairy roots of *B. rapa* and *B. napus* at different time points (0.5, 1, 2, and 4 h) of cytokinin (5 µM BAP) treatment. Means ±SE are shown in the plots. Asterisks indicate statistical significance (***P<0.001, **P<0.01, and *P<0.05, Dunnett’s test). (C) Representative images of *B. rapa* and *B. napus* hairy root tips treated with DMSO and BAP throughout the treatment period, showing the measured fluorescent signal intensities in a single root (top) and the corresponding image analyzed by IMARIS software (below). Scale bars represent 40 µm.

To validate these findings, we utilized the hairy root transformation system (Jedlickova *et al.*, 2022) to introduce the cytokinin-responsive reporter (*TCSv2*:*3×VENUS*) developed in *A. thaliana* by Steiner *et al.* (2020) into *Brassica* species. *TCSv2* incorporates concatemerized RRB-binding motifs with a distinct arrangement (Fig. 8A) that enhances sensitivity when compared with the original version of the TCS reporter (Zurcher *et al.*, 2013). Compared with a mock-treated control, a significant increase in the relative fluorescence intensity was observed after 30 min and 1 h of the cytokinin treatment in the hairy roots of *B. napus* and *B. rapa*, respectively, carrying *TCSv2:3×VENUS* (Fig. 8B, C).

Taken together, our results strongly suggest that similarly to Arabidopsis, the *Brassica* RRBs recognize conserved *cis*-regulatory regions to mediate the cytokinin-induced transcriptional activation of *Brassica RRA* genes and possibly other cytokinin-responsive genes within the *Brassica* genomes.

### Cold stress stimulates *RRA* expression in the *Brassicaceae* family

**Fig. 9.**
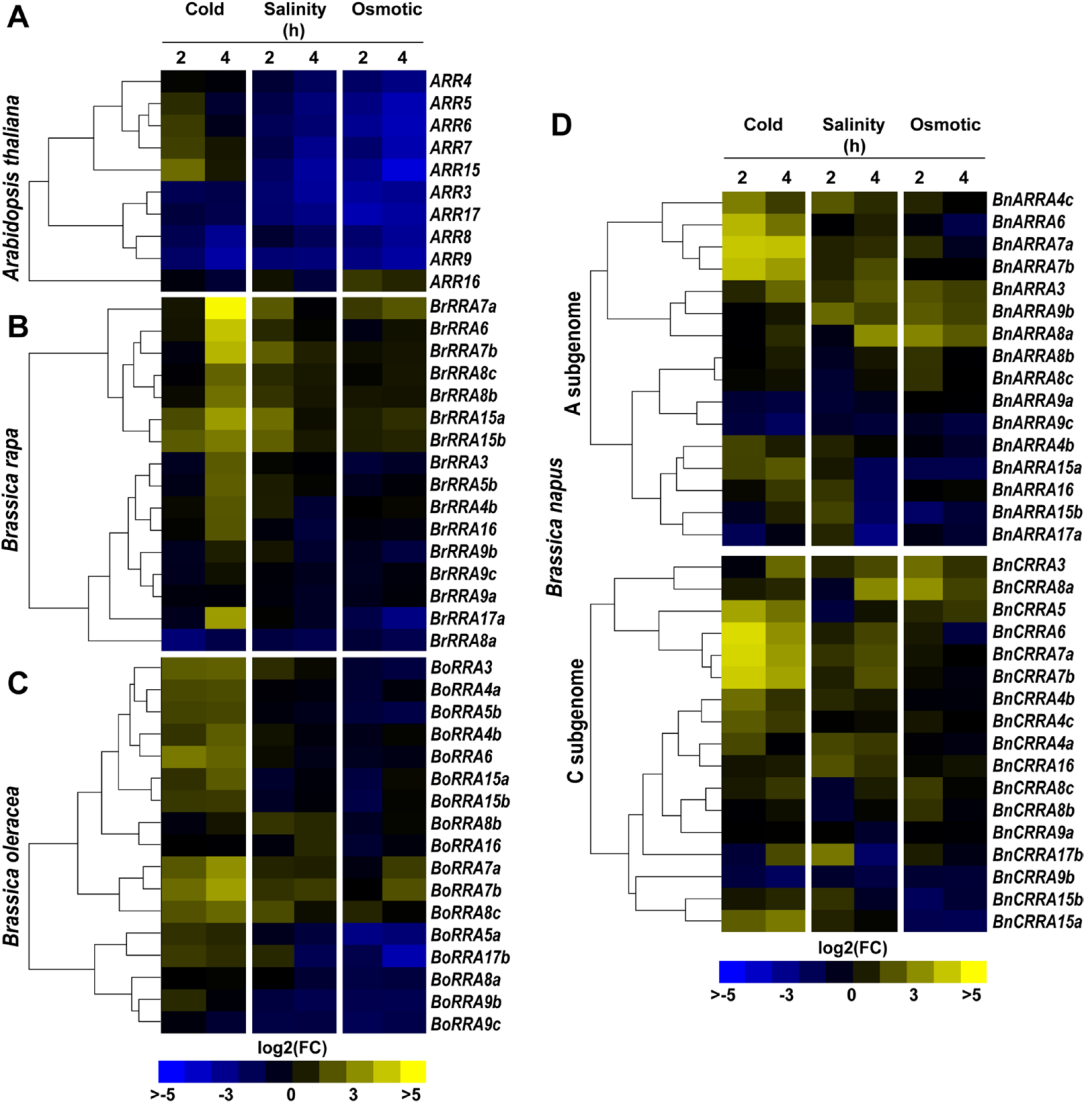
*Arabidopsis thaliana* and *Brassica RRA* genes respond to abiotic stress. Heat maps depicting the expression pattern of *RRA* genes in 1-week-old seedlings of (A) *A. thaliana*, (B) *B. rapa*, (C) *B. oleracea*, and (D) *B. napus* under cold (4 °C), salinity (250 mM NaCl), and osmotic stress (300 mM mannitol) conditions for 2 h and 4 h (see the Materials and methods). The expression data are presented as log2 fold change normalized to the mock treatment by the delta-delta Ct (Pfaffl, 2004); for the color code see the key.

To assay the possible stress-related regulation of *RRA* genes within the *Brassicaceae* family, the expression profiles of the 66 selected *Brassica RRA* genes and the 10 *A. thaliana RRA* genes were investigated after exposure to cold (4 °C), salinity (250 mM NaCl), and osmotic stress (300 mM mannitol). In *A. thaliana*, cold stress rapidly (within 2 h after the stress application) up-regulated the expression of several *RRA* genes, in particular *ARR6*, *ARR7*, and *ARR15*. However, the cold-induced up-regulation was transient, and the expression of up-regulated *RRA* genes returned to basal levels after 4 h of cold exposure. In contrast, we observed gradual repression of *ARR3*, *ARR8*, *ARR9*, *ARR16*, and *ARR17* at 2 h and 4 h of the cold stress application (Fig. 9A; Supplementary Table S6). In *B. rapa*, greater numbers of *RRA* genes were up-regulated in the response to cold, although the induction was delayed when compared with *A. thaliana.* Most *BrRRA* genes, except for the non-responsive *BrRRA8a*, *BrRRA9a*, *BrRRA9b*, and *BrRRA9c*, exhibited up-regulation after 4 h of cold exposure. *BrRRA15a* and *BrRRA15b* showed an earlier response, being up-regulated after 2 h of chilling, and remained activated for the 4 h of the treatment (Fig. 9B; Supplementary Table S6). Also in *B. oleracea*, most of the *BoRRA* genes were up-regulated by cold stress. Similarly to *A. thaliana*, the response was evident early (2 h) during cold exposure; however, compared with the transient up-regulation seen in the cold-responsive *A. thaliana RRA* genes, the up-regulation of *BoRRA* genes lasted the entire 4 h of treatment. This response pattern was observed for *BoRRA6*, *BoRRA7a*, *BoRRA7b*, *BoRRA15a*, and *BoRRA15b* (Fig. 9C; Supplementary Table S6). Also in *B. napus*, we observed prompt up-regulation of *RRA* genes lasting for the 4 h of the cold treatment. This type of response was apparent for homologs of *ARR6* (*BnARRA6* and *BnCRRA6*), *ARR7* (*BnARRA7a*, *BnARRA7b*, *BnCRRA7a*, and *BnCRRA7b*), and *ARR15* (*BnARRA15a* and *BnCRRA15b*). Several other *BnRRA* genes, including homologs of *ARR3*, *ARR4*, *ARR5*, *ARR8*, and *ARR17*, were also up-regulated by cold, but with variable kinetics (Fig. 9D; Supplementary Table S6).

In summary, several *RRA* genes are up-regulated in response to cold stress in the *Brassicaceae* family, albeit with slightly different kinetics. *ARR6*, *ARR7*, *ARR15*, and their *Brassica* homologs appear to represent the core of the common cold-responsive transcriptional signature among the *RRA* genes.

### Salinity and osmotic stress lead to contrasting expression of *A. thaliana* and *Brassica RRA* genes

Compared with cytokinin and cold treatment, the majority of *A. thaliana RRA* genes exhibited down-regulation after exposure to salinity and osmotic stress, except for *ARR16*, which showed up-regulation after 2 h of salinity stress (Fig. 9A; Supplementary Table S6). In contrast, several *BrRRA* genes were up-regulated after 2 h of salinity exposure, particularly the homologs of *ARR6* (*BrRRA6*), *ARR7* (*BrRRA7a*, *7b*), and *ARR15* (*BrRRA15a* and *BrRRA15b*). However, only *BrRRA7b* displayed up-regulation when exposed to osmotic stress (Fig. 9B; Supplementary Table S6). In *B. oleracea*, homologs of *ARR7* (*BoRRA7b* and *BoRRA7c*) along with *BoRRA8b* and *BoRRA8c* were up-regulated after 2 h of salinity treatment, and this effect persisted up to 4 h, except for *BoRRA8b*. In response to osmotic stress, only homologs of *ARR7* (*BoRRA7a* and *BoRRA7b*) were up-regulated after 4 h of treatment (Fig. 9C; Supplementary Table S6). In contrast to their diploid ancestors, there were more *RRA* genes in *B. napus* that were induced by salinity and/or osmotic stress after either 2 h or 4 h of stress exposure. These included *BnARRA3*, *BnARRA7a*, *BnARRA7b*, *BnARRA8a*, *BnARRA8b*, *BnARRA8c*, and *BnARRA9b* in the A genome and all *RRA* genes from the C-genome, except *BnARRA9a* and *BnARRA9b.*

Overall, *RRA* genes in *Brassicaceae* are regulated by salt and osmolarity stresses, displaying various types (up- versus down-regulation) and dynamics of the response. Compared with *A. thaliana RRA* genes being mostly down-regulated, all tested *Brassica* crops exhibited up-regulation of *RRA* genes in the presence of not only cytokinins but also of abiotic stresses. Similar to the cold treatment, homologs of *ARR7* and *ARR15* appear to be a sensitive readout of the response to salinity and high osmolarity in both diploid *Brassica* species, *B. rapa* and *B. oleracea*. However, particularly in *B. napus*, the response to these stress types seems to be more general, involving a larger number of *RRA* genes.

### Cytokinin-responsive reporter *TCSv2* as a sensitive tool for studying early stress responses

**Fig. 10.**
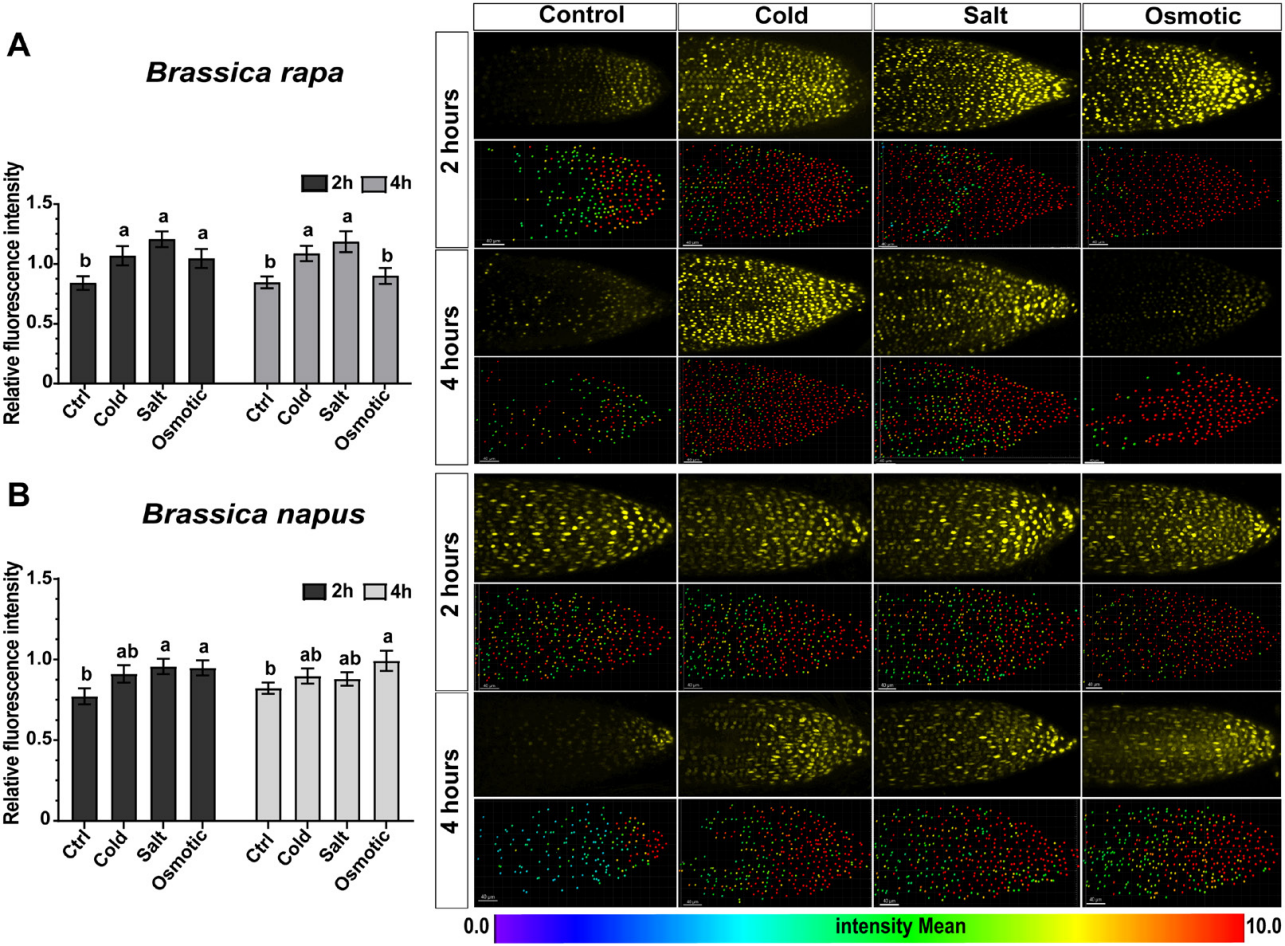
The Arabidopsis *TCSv2:3×VENUS* cytokinin reporter (Steiner *et al.*, 2020) in *B. rapa* and *B. napus* is sensitive to early stress response. Comparison of the relative fluorescence intensity of the *TCSv2:3×VENUS* cytokinin reporter in control and abiotic stress-treated [cold (4 °C), salinity (250 mM NaCl), and osmotic stress (300 mM mannitol)] hairy roots of *B. rapa* (A) and *B. napus* (B) at different time points (2 h and 4 h; see the Materials and methods). Means ±SE are shown in the plots. The different letters indicate variable groups with statistically significant differences (*P*<0.05, Tukey’s HSD). Representative images of *B. rapa* (A) and *B. napus* (B) hairy root tips under control and abiotic stress throughout the treatment period, showing the measured fluorescent signal intensities in a single root (top) and the corresponding image analyzed by IMARIS software (below). Scale bars represent 40 µm.

To elucidate the molecular mechanism of stress-induced *RRA* up-regulation in *Brassica* sp., we investigated the response of the *TCSv2::3×VENUS* reporter to various abiotic stresses in *B. rapa* and *B. napus* root tips (Fig. 10). Notably, under cold, salt, and osmotic stress conditions, in *B. rapa TCSv2*-driven VENUS exhibited a significant increase of intensity 2 h and 4 h post-treatment, with the exception of osmotic stress, showing significant up-regulation only after 2 h of the treatment (Fig. 10A). Compared with that, *B. napus* showed an induction of VENUS intensity by all stress treatments after 2 h, with salt and osmotic stress showing significant differences compared with the control. However, no significant differences were observed between treatments and control conditions at 4 h of treatment, except for osmotic stress (Fig. 10B). In conclusion, our observations clearly show sensitivity of the (RRB-regulated) cytokinin reporter system in *Brassica* species to abiotic stress, emphasizing its utility in discerning early stress responses in crops.

### Cytokinins contribute to the cold stress-induced up-regulation of *RRA* genes in *Brassicaceae*

**Fig. 11.**
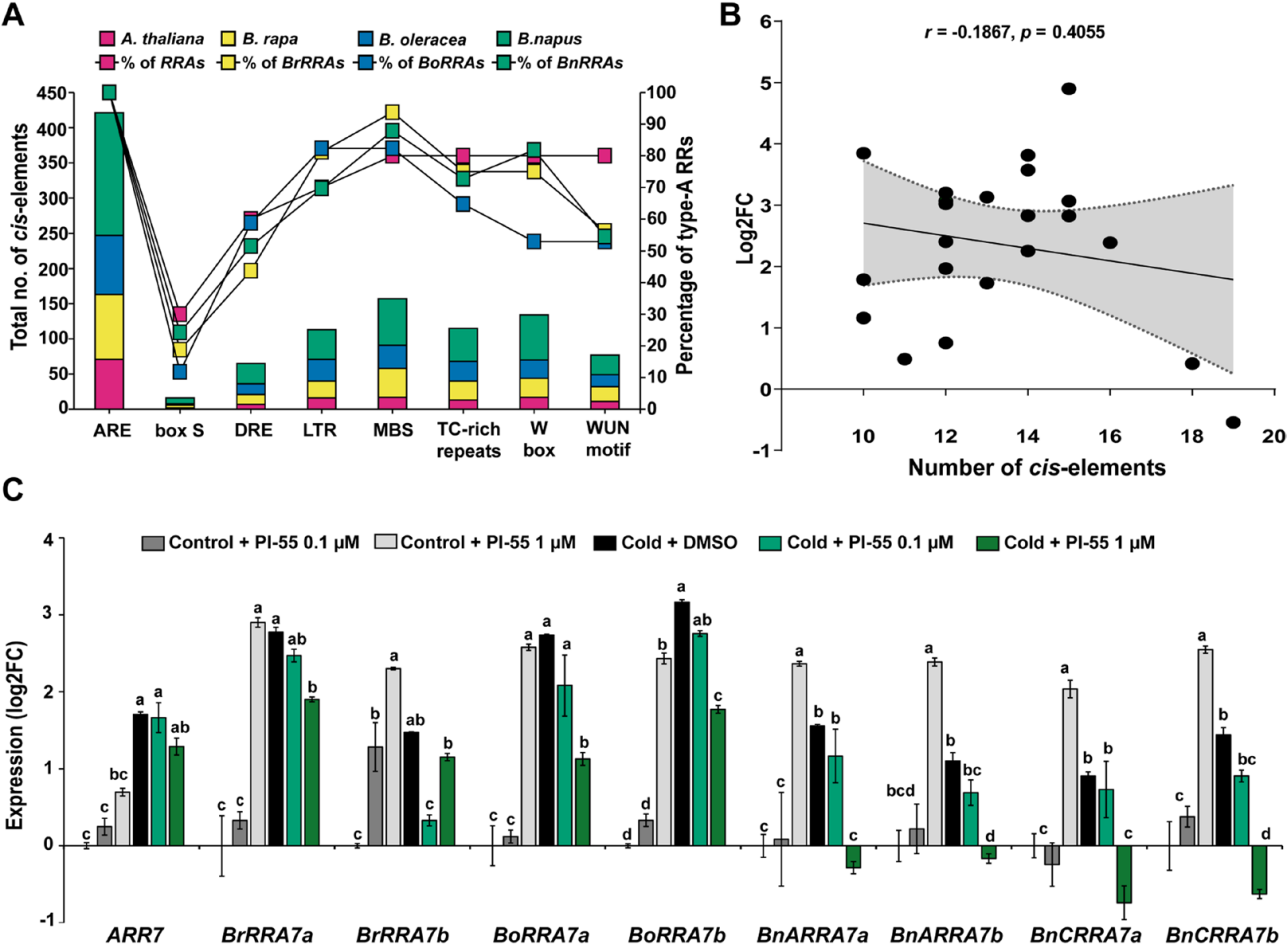
Role of cytokinins dominates over environmental stress-related *cis*-elements in cold-induced *RRA* up-regulation in *Brassica* species. (A) Comparison of the number of environmental stress-related *cis*-elements identified using the PlantCARE databases (Lescot *et al.*, 2002) in the promoter regions of *A. thaliana* and *Brassica RRA* genes along with the percentage of *RRA* genes where these *cis*-elements were found. (B) Pearson correlation (with 95% confidence interval, shadowed part) between the transcriptional response of cold-responsive *A. thaliana* and *Brassica RRA* genes after 4 h of cold treatment and the number of environmental *cis*-elements present in their promoter regions. (C) Expression of *ARR7* and its homologs after incubation of 1-week-old seedlings in medium supplemented with either DMSO or the cytokinin antagonist PI-55 (0.1 µM/1 µM) and exposure for 4 h to either cold or control conditions. The expression data are presented as log2 fold change double normalized by the delta-delta Ct (Pfaffl, 2004) (means ± SE) to the corresponding housekeeping gene (see the Materials and methods) and the control. The different letters indicate variable groups with statistically significant differences (*P*<0.05, Tukey’s HSD).

Our gene expression data show the regulation of *RRA* genes by abiotic stresses. Utilizing the online database and PlantCARE portal (Lescot *et al.*, 2002), several environmental stress-related *cis*-elements were identified in all the promoter sequences of *A. thaliana RRA* genes, 16 *BrRRA* genes and *BnARRA* genes, and 17 *BoRRA* genes and *BnCRRA* genes (Fig. 11A; Supplementary Table S7). However, the correlation tests between the number of identified stress-related *cis*-elements and the expression of cold-responsive *ARR6*, *ARR7*, *ARR15*, and their *Brassica* homologs after cold exposure did not yield any statistically significant results (Fig. 11B; Supplementary Fig. S6). In an alternative approach, we searched the DAP-seq data (Bartlett *et al.*, 2017) to find TFs with potential binding sites in *A. thaliana RRA* promoters. We found six such TFs (AT2G28810, AT3G52440, AT5G56840, ATHB25, ATHB23, and ATHB34); however, the significance of enrichment of their binding sites in the stress-responsive *A. thaliana* and *Brassica RRA* genes was low (Supplementary Tables S8, S9). Altogether, our data do not provide any solid evidence supporting the role of the identified stress-related *cis*-regulatory elements in the control of *RRA* gene expression within the *Brassicaceae* family.

To assess the possible involvement of cytokinins in the cold stress-mediated up-regulation of *RRA* genes, we tested the cold response of *ARR7* and its *Brassica* homologs in the presence of the anticytokinin (cytokinin signaling inhibitor) PI-55. PI-55 was demonstrated to inhibit the activation of the MSP signaling cascade by competing with cytokinin binding to the CHASE domain of AHKs (Spichal *et al.*, 2009). Under control conditions, treatment with PI-55 led to the induction of all tested *RRA* genes, probably due to its previously reported weak cytokinin activity (Spichal *et al.*, 2009). However, when applied under low-temperature conditions, PI-55 was able to reduce the cold-induced up-regulation of *ARR7* and its *Brassica* homologs. However, it should be pointed out here that although a similar trend was apparent in all species tested (except for *BrRRA7b*), the effect was statistically significant only in *B. oleracea* and was particularly strong in *B. napus*, where the presence of 1 µM PI-55 completely abolished the up-regulation of cold-induced *B. napus ARR7* homologs and led to the drop of gene expression even under the control levels (Fig. 10C).

In conclusion, our findings suggest the existence of a cytokinin-dependent mechanism that contributes to the activation of several *RRA* genes in the response to cold stress.

## Discussion

### *Brassica* and *A. thaliana RRA* genes reveal a close evolutionary relationship

We unearthed a total of 78 putative RRAs within the genomes of *B. oleracea*, *B. rapa*, and *B. napus*. Our investigation not only validates the prior identification of certain RRAs reported in the genomes of *B. rapa* and *B. oleracea* but also introduces novel candidates, expanding our understanding of the MSP regulatory landscape in these *Brassica* species from an evolutionary perspective.

Three rounds of whole-genome duplications (WGDs) took place in *Brassicaceae* after its lineage diverged from monocots but prior to the further divergence within the family (Moghe *et al.*, 2014). Kalteneger *et al*. (2018) proposed the presence of two *RRA* copies (possibly resulting from the ancient ζ or ε WGD event) in the last common ancestor before the divergence of monocots and dicots. Four of the five paralogous RRA pairs (ARR6/ARR5, ARR15/ARR7, ARR8/ARR9, and ARR17/ARR16) (Kaltenegger *et al.*, 2018) probably originated through the later α WGD event dated to ~47 million years ago (Mya). More recently (~25 Mya), an α' whole-genome triplication (WGT) event took place in the *Brassica* ancestor after the divergence from the Arabidopsis lineage (Lysak *et al.*, 2005; Town *et al.*, 2006; Yang *et al.*, 2006; Wang *et al.*, 2011), leading to the formation of 20 *RRA* genes in both *B. oleracea* and *B. rapa*.

The allotetraploid *B. napus* is a result of interspecific hybridization between *B. rapa* and *B. oleracea* (Nagaharu and Nagaharu, 1935; Zhang *et al.*, 2016). In accordance with that, the 20 *BnARRA* genes identified in the A subgenome and 18 *BnCRRA* genes found in the C subgenome exhibit notable similarity and are mostly syntenic with their counterparts in the *B. rapa* and *B. oleracea* genomes, respectively. Considering the close evolutionary relationships, we used the well-established *A. thaliana RRA* genes (*ARR* genes) as a reference and numbered the newly identified *B. napus RRA* gene according to their (putative) *A. thaliana* orthologs. For the sake of consistency, we extended this type of numbering to the newly identified *BoRRA* genes as well as to the previously described *BrRRA* and *BoRRA* genes (Kaltenegger *et al.*, 2018). We believe that this nomenclature type facilitates comparative analyses within the large gene families of closely related species including the description of gene structure or expression profiles, as we demonstrated in our work. Obviously, different reference species must be used for the monocotyledonous plants that evolved the individual components of (not only) MSP signaling separately (Kaltenegger *et al.*, 2018).

### Homologs of *ARR3*, *ARR6*, and *ARR16* are under evolutionary pressure against multiplication during *Brassicaceae* evolution

Gene or genome multiplication is an indispensable feature of plant evolution, and gene loss is a frequent fate of newly multiplicated genes (Lynch and Conery, 2000). More specifically, the majority of orthologous groups (~70%) in the common progenitor of recent *Brassicaceae* species *Raphanus raphanistrum* and *B. rapa* experienced losses after the WGT (Moghe *et al.*, 2014). Interestingly, genes encoding individual MSP components (i.e. sensor HKs, HPts, and RRs) differ in the extent of gene loss and preservation during evolution. While in the case of HKs, gene loss is a dominant feature, response regulators, particularly RRAs are mostly preserved after WGDs (Kaltenegger *et al.*, 2018).

In this context, we have rather surprisingly identified homologs of *ARR3*, *ARR6*, and *ARR16* as singletons in both *B. rapa* and *B. oleracea* (Fig. 3), suggesting evolutionary pressure against the multiplication of those genes. The presence of two copies of the *ARR3*, *ARR6*, and *ARR16* homologs in *B. napus* (a single copy in each subgenome) might be explained by the recency of the interploidization event. We confirmed the singleton status of *ARR3*, *ARR6*, and *ARR16* orthologs also in other *Brassicaceae* species including diploid *Camelina sativa* and a single copy per subgenome in the allotetraploid *Brassica juncea* (Supplementary Figs S7, S8). The ability of the gene duplication to be retained seems to be associated with sequence and expression divergence, leading to functional diversification (Moghe *et al.*, 2014). In our cytokinin and abiotic stress response assays, we did not observe any strong expression specificity of *ARR3*, *ARR6*, or *ARR16* and their *Brassica* orthologs, potentially explaining the singleton status of those genes. In *A. thaliana*, some of the *RRA* genes were shown to play specific roles in controlling plant growth and development that cannot be explained solely by their functions as redundant cytokinin primary response genes and negative regulators of MSP signaling. To name a few, the ethylene-inducible *ARR3* regulates RAM size (Zdarska *et al.*, 2019) and is involved in the cytokinin-independent control over circadian rhythms (Salome *et al.*, 2006). ARR6 mediates a negative interaction between abscisic acid and MSP signaling (Wang *et al.*, 2011; Huang *et al.*, 2017), plays a role in the CLE peptide-mediated inhibition of protoxylem formation (Kondo *et al.*, 2011), and regulates pathogen immune response by controlling cell wall composition (Bacete *et al.*, 2020). Finally, spatial-specific expression of *ARR16* and *ARR17* regulates the hydrotropic bending of the root (Chang *et al.*, 2019), and controls stomata formation (Vaten *et al.*, 2018) and leaf growth (Efroni *et al.*, 2013). Thus, *ARR3*, *ARR6*, and *ARR16* seem to mediate several key regulatory roles, which might be sensitive to gene dosage. To what extent the *Brassica* homologs of those genes play similar regulatory roles and whether this explains the observed negative selection, however, remains to be clarified.

### Cytokinins contribute to abiotic stress-mediated induction of a subset of *RRA* genes

The *A. thaliana RRA* genes were originally described as cytokinin primary response genes, being rapidly (in the order of minutes) induced by exogenous cytokinin treatment (D’Agostino *et al.*, 2000). Here, we categorized the *RRA* genes based on the kinetics of their cytokinin response into seven categories: (i) fast, (ii) fast-transient, (iii) down-regulated, (iv) later, (v) later transient, (vi) other, and (vii) no response. The corresponding transcriptional dynamics may reflect certain specificity within MSP signaling (Pekarova *et al.*, 2016), with a possible impact on the downstream molecular network underlying the cytokinin cellular responses (Skalak *et al.*, 2019). The proportion of individual *RRA* categories varied among tested species, with categories (iv) later, (v) later transient, (vi) other, and (vii) no response being specific for *Brassica* sp. However, a subset of *RRA* genes, including homologs of *ARR5*, *ARR7*, and *ARR15* [all belonging to class (i) fast] exhibited comparable cytokinin responses in all the tested species. This observation, together with a high level of conservation of the DNA-binding GARP domain of RRBs and the cytokinin responsiveness of the *TCSv2* reporter in *B. rapa* and *B. napus*, implies that *RRA* genes may share common features and functions within the *Brassicaceae* family. Interestingly, we observed that a subset of cytokinin-responsive *RRA* genes of the category (i) fast constitutes a core of the abiotic stress-responsive *RRA* genes. While homologs of *ARR6*, *ARR7*, and *ARR15* were cold responsive, *RRA* genes similar to *ARR7* and *ARR15* (together with other *RRA* genes, particularly in *B. napus*) seem also to be involved in the response to salinity and high osmolarity in all the tested *Brassica* species, suggesting the existence of a common regulatory mechanism. This conclusion is also supported by the rapid induction of cytokinin reporter *TCSv2* by all tested stress conditions. As the *TCSv2* activation is solely RRB dependent, these data clearly support the involvement of MSP signaling in the abiotic stress response of *Brassica* crops. The TCS-based reporters were previously shown to reliably reflect the cytokinin signaling output in crops such as rice and tomato (Tao *et al.*, 2017; Steiner *et al.*, 2020). While the environmental conditions such as shade or osmotic stress significantly regulate TCS reporter activity in Arabidopsis (Novák *et al.*, 2015; Rowe *et al.*, 2016; Chang *et al.*, 2019), no observation of stress-dependent regulation of the TCS system has been studied in crops so far. Thus, our results open up a new path facilitating further studies on the dynamics of signal transduction and stress adaptation in crops.

Our finding on the contribution of cytokinin signaling to the cold-mediated regulation of *ARR7* and its *Brassica* homologs is in line with this hypothesis. (A)biotic stress has been shown to control endogenous hormone levels, including cytokinins, at the level of both biosynthesis and metabolism (Skalak *et al.*, 2021, and references therein). This implies that stress-induced up-regulation of endogenous cytokinin levels might be a part of the cold (and probably other abiotic stress) response in *Brassicaceae*, thus further substantiating the proposed role of plant hormones as a regulatory interface between environmental conditions and intrinsic regulatory pathways controlling individual processes of plant growth and development (Ramireddy *et al.*, 2014; Landrein *et al.*, 2018; Cortleven *et al.*, 2019; Skalak *et al.*, 2021; Yamoune *et al.*, 2021; Abualia *et al.*, 2022; Waadt *et al.*, 2022; Taleski *et al.*, 2023).

### Conclusions and future outlines

In summary, our work sheds light on the evolutionary relationships of MSP signaling within the *Brassicaceae* family. We provide a complete list of the *RRA* genes and their partial molecular characterization in the allotetraploid *B. napus* but also in its parental species, *B. rapa* and *B. oleracea.* That includes a novel classification reflecting the kinetics of their cytokinin-dependent transcriptional regulation. The conserved occurrence of *ARR3*, *ARR6*, and *ARR16* and their orthologs as singletons in diploid members of the *Brassicaceae* family (*A. thaliana*, *C. sativa*, *B. rapa*, and *B. oleracea*) and a single copy per subgenome in allotetraploids *B. napus* and *B. juncea* implies the existence of gene-specific negative selection, possibly based on functional importance and preventing gene multiplication. Several of the *RRA* genes exhibited conserved expression patterns in response to cytokinin and abiotic stresses, implying the presence of common regulatory elements. Our data suggest that cold-mediated induction of *RRA* genes demands canonical cytokinin signaling in all tested *Brassica* species, thus emphasizing the importance of cytokinin-regulated MSP in abiotic stress responses. These findings contribute to a nuanced comprehension of the pivotal role of *RRA* genes in plant stress responses and open up novel avenues for further investigation to uncover the intricate mechanisms guiding plant growth and adaptation, with high potential for applied research. In this respect, the functional characterization of *RRA* genes, although challenging considering the redundancy previously observed in Arabidopsis (To *et al.*, 2004), will be the next important goal in our efforts to elucidate their role in the abiotic crop response.

## Supplementary data

The following supplementary data are available at *JXB* online.

Table S1. Gene and protein information on type-A response regulators from *A. thaliana*.

Table S2. Gene and protein information for *Brassica RRA* genes.

Table S3. List of primers used in the study.

Table S4. Relative expression of 1-week-old seedlings of *A. thaliana*, *B. rapa*, *B. oleracea*, and *B. napus* after cytokinin treatment.

Table S5. Arabidopsis-like cytokinin-responsive *cis*-elements identified in the promoter regions of *A. thaliana* and *Brassica RRA* genes.

Table S6. Relative expression of 1-week-old seedlings of *A. thaliana*, *B. rapa*, *B. oleracea*, and *B. napus* after exposure to abiotic stress treatment.

Table S7. Environmental stress-related *cis*-elements identified in the promoter regions of the type-A response regulators in *A. thaliana*, *B. rapa*, *B. oleracea*, and *B. napus*.

Table S8. Enrichment of stress-responsive transcription factors identified in the promoter regions of stress-responsive *RRA* genes in *A. thaliana*.

Table S9. Enrichment of stress-responsive transcription factors identified in the promoter regions of stress-responsive *BrRRA*, *BoRRA*, and *BnRRA* genes.

Fig. S1. Phylogenetic relationship of type-A response regulators in *A. thaliana* and *B. rapa*.

Fig. S2. Phylogenetic relationship of type-A response regulators in *A. thaliana* and *B. oleracea*.

Fig. S3. Phylogenetic relationship of type-A response regulators in *A. thaliana* and *B. napus* (A and C subgenome).

Fig. S4. The mean expression levels of *B. rapa* and *B. oleracea RRA* genes.

Fig. S5. The DNA-binding domains of type-B RRs are conserved in the *Brassicaceae*.

Fig. S6. Stress-responsive elements do not seem to control the expression of cold-responsive RRAs in Arabidopsi*s* and *Brassica* sp.

Fig. S7. Phylogenetic relationship of RRAs in *A. thaliana* and *Brassica juncea*.

Fig. S8. Phylogenetic relationship of RRAs in *A. thaliana* and *Camelina sativa.*

erae335_suppl_Supplementary_Tables_S1-S9

erae335_suppl_Supplementary_Figures_S1-S8

## Data Availability

All data supporting the findings of this study are available within the paper and its supplementary data published online.
