# Supplementary material for: Primary multistep phosphorelay activation comprises both cytokinin and abiotic stress responses: insights from comparative analysis of Brassica type-A response regulators
Source: J Exp Bot. 2024 Aug 22;75(20):6346–68. doi: 10.1093/jxb/erae335 (PMC11523033; doi:10.1093/jxb/erae335)
Supplement: erae335_suppl_Supplementary_Figures_S1-S8 [file erae335_suppl_supplementary_figures_s1-s8.pdf]

Supplementary data

1 Supplementary Figures

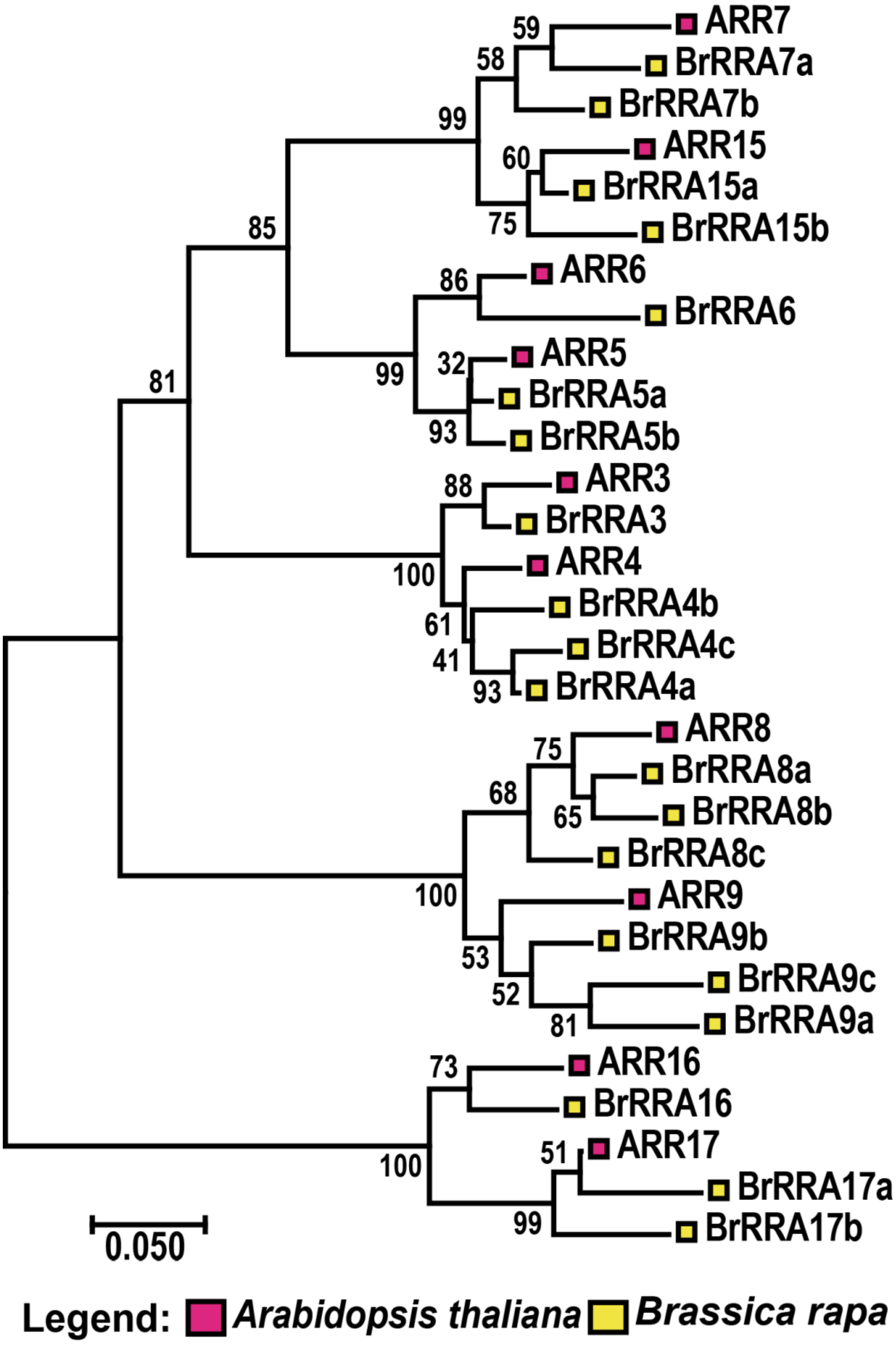

Supplementary Figure S1. Phylogenetic relationship of type-A response regulators in *A. thaliana* and *B. rapa*. The unrooted tree is constructed based on the similarity of the RRA protein sequences using the Neighbor-Joining method. The bar indicates the relative divergence of the examined sequences. Individual species are distinguished by the color of the box (dark pink for *A. thaliana*, and yellow for *B. rapa*).

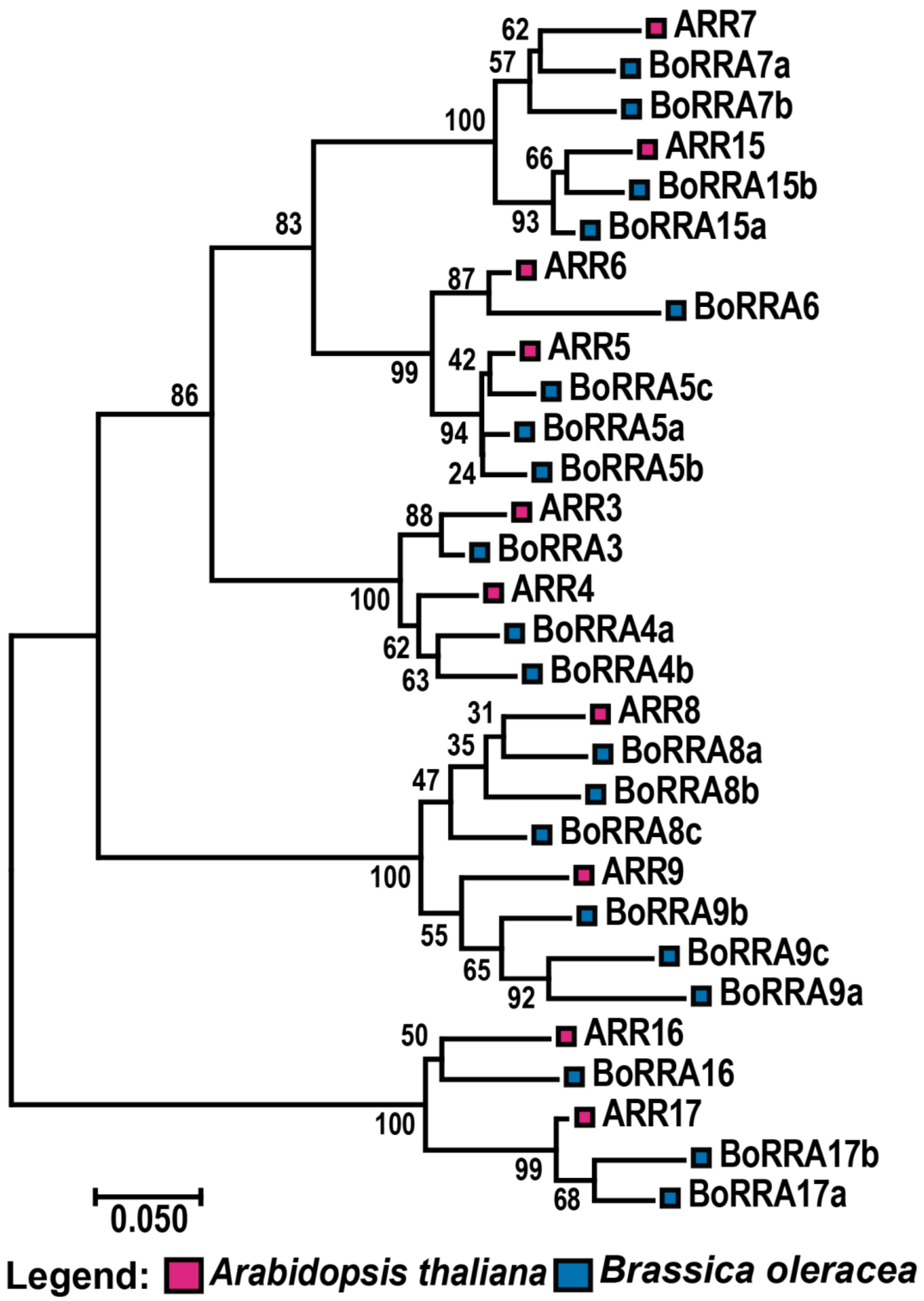

Supplementary Figure S2. Phylogenetic relationship of type-A response regulators in *A. thaliana* and *B. oleracea*. The unrooted tree is constructed based on the similarity of the RRA protein sequences using the Neighbor-Joining method. The bar indicated the relative divergence of the examined sequences. Individual species are distinguished by the color of the box (dark pink for *A. thaliana*, and blue for *B. oleracea*).

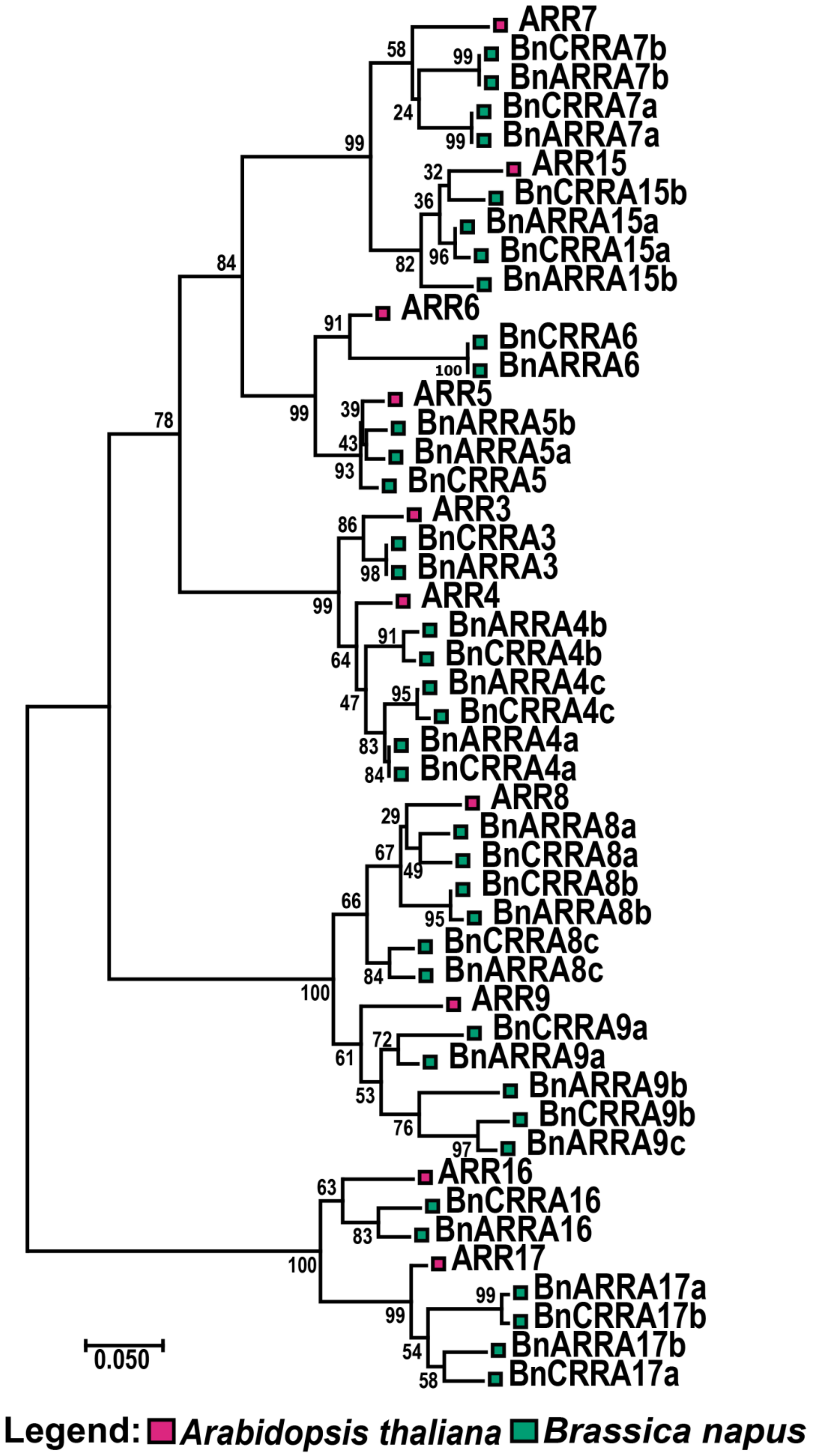

Supplementary Figure S3. Phylogenetic relationship of type-A response regulators in A. thaliana and B. napus (A and C subgenome). The unrooted tree is constructed based on the similarity of the RRA protein sequences using the Neighbor-Joining method. The bar indicated the relative divergence of the examined sequences. Individual species are distinguished by the color of the box (dark pink for *A. thaliana*, and green for *B. napus*).

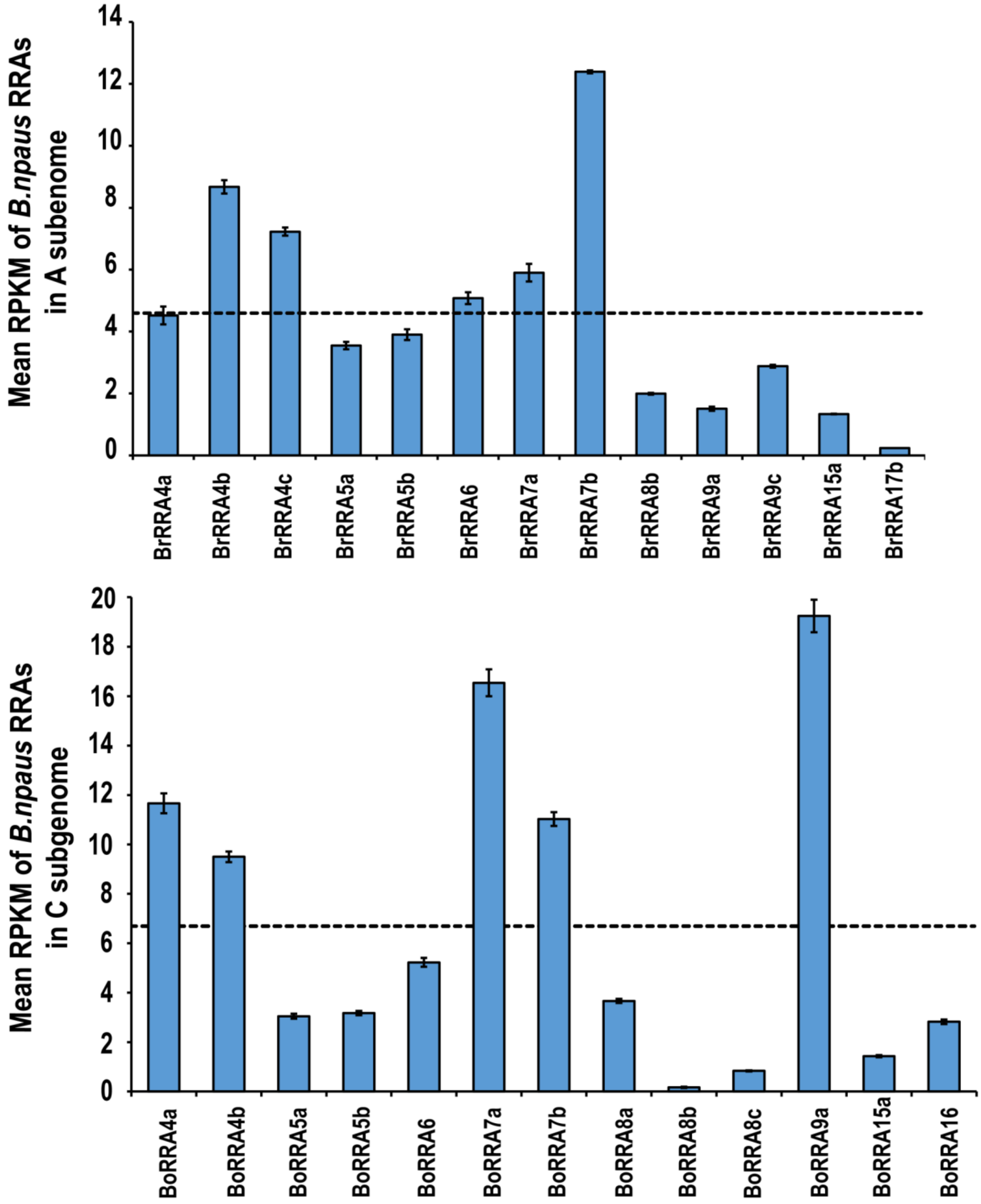

Supplementary Figure S4. The mean expression levels of *BrRRAs (B. rapa RRAs)* and *BoRRAs (B. oleracea RRAs*). The expression levels are shown as Reads Per Kilobase of transcript per Million mapped reads (RPKM; mean ± SE) of individual RRAs from the transcriptomes of Brassica napus (A and C subgenome) cultivars as determined in the Renewable Industrial Products from Rapeseed (RIPR) diversity panel (Havlickova et al., 2018). The dotted lines indicate the average RPKM values.

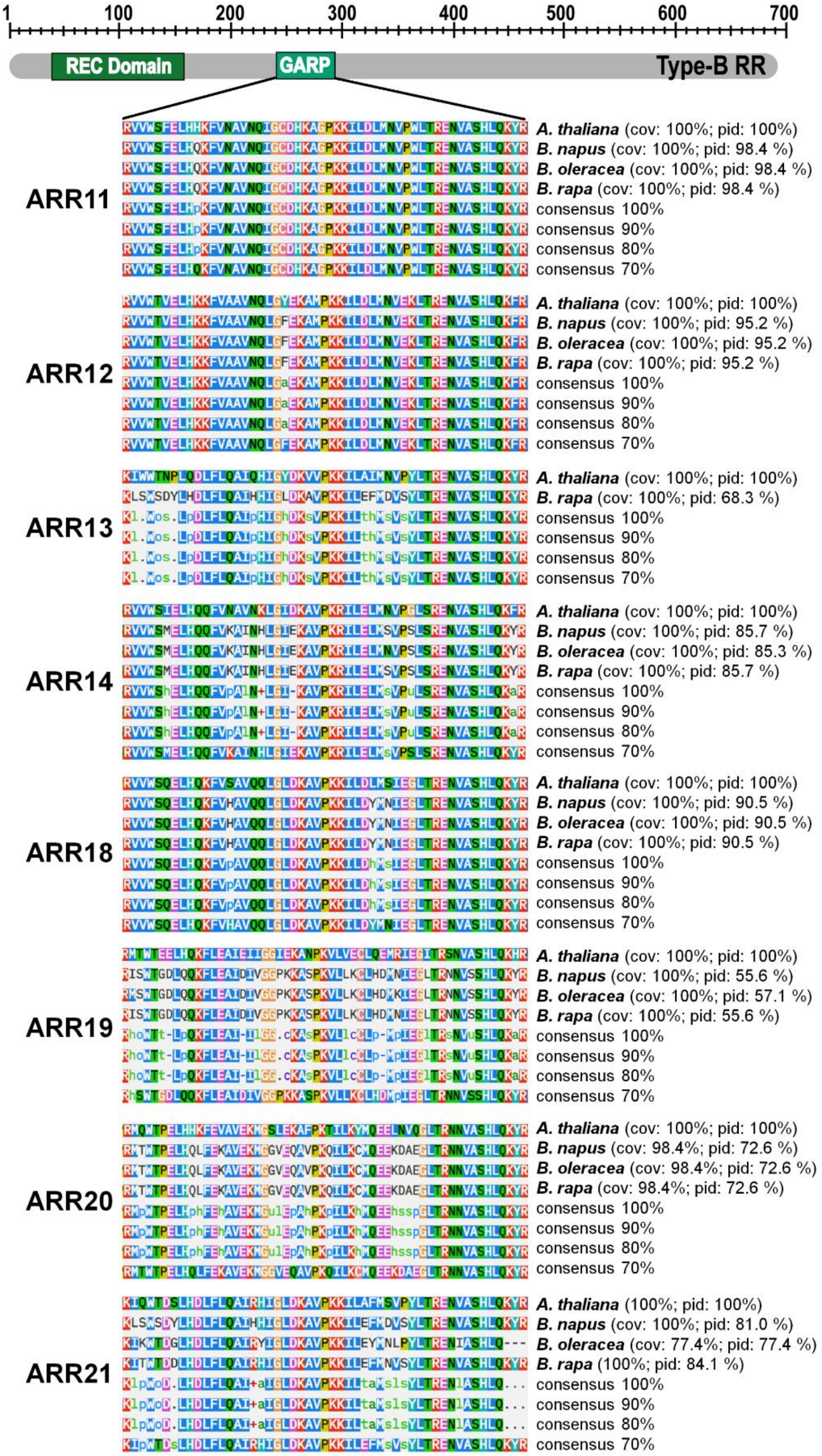

Supplementary Figure S5. The DNA binding domains are of type-B RRs are conserved in the Brassicacea. Multiple alignments of GARP-like DNA binding domain of the type-B RRs proteins (i.e., ARR11, ARR12, ARR13, ARR14, ARR18, ARR19, and ARR20) from *A. thaliana* (Ath), and assayed Brassica species [*B. napus* (Bna), *B. oleracea* (Bol), and *B. rapa* (Bra)]. Conserved amino acids are highlighted, and the coverage (cov) and percent identity (pid) are indicated as percentages. The consensus sequence is also shown (consensus/70%-100%). The CLUSTAL color scheme was used to color the alignment, reflecting the physicochemical properties of amino acids (Kunzmann et al., 2020).

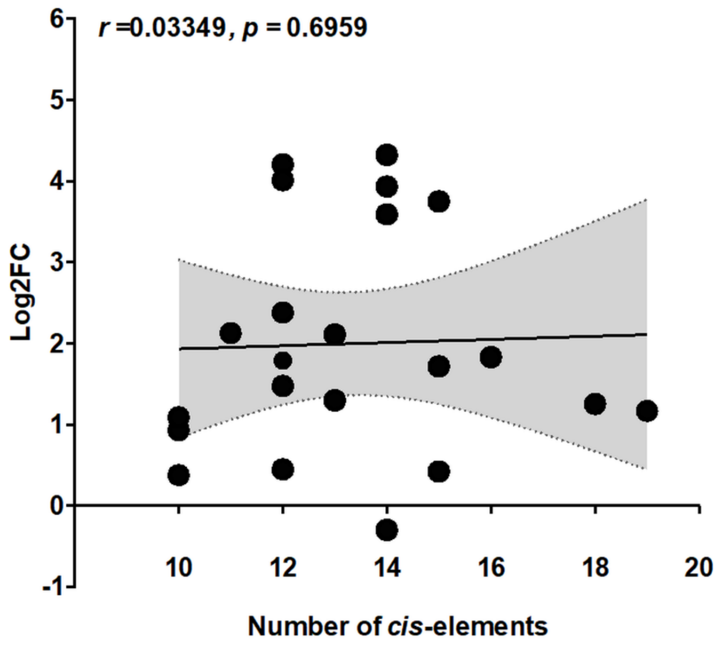

Supplementary Figure S6. Stress-responsive elements do not seem to control the expression of cold-responsive RRAs in *Arabidopsis* and *Brassica sp*.. Pearson correlation (with 95% confidence intervals, shadowed part) between the gene expression of cold-responsive *A. thaliana* and *Brassica* RRAs after 2 hours of cold treatment and the number of environmental stress-related *cis*-elements identified using the PlantCARE databases (Lescot et al., 2002) in their promoter regions.

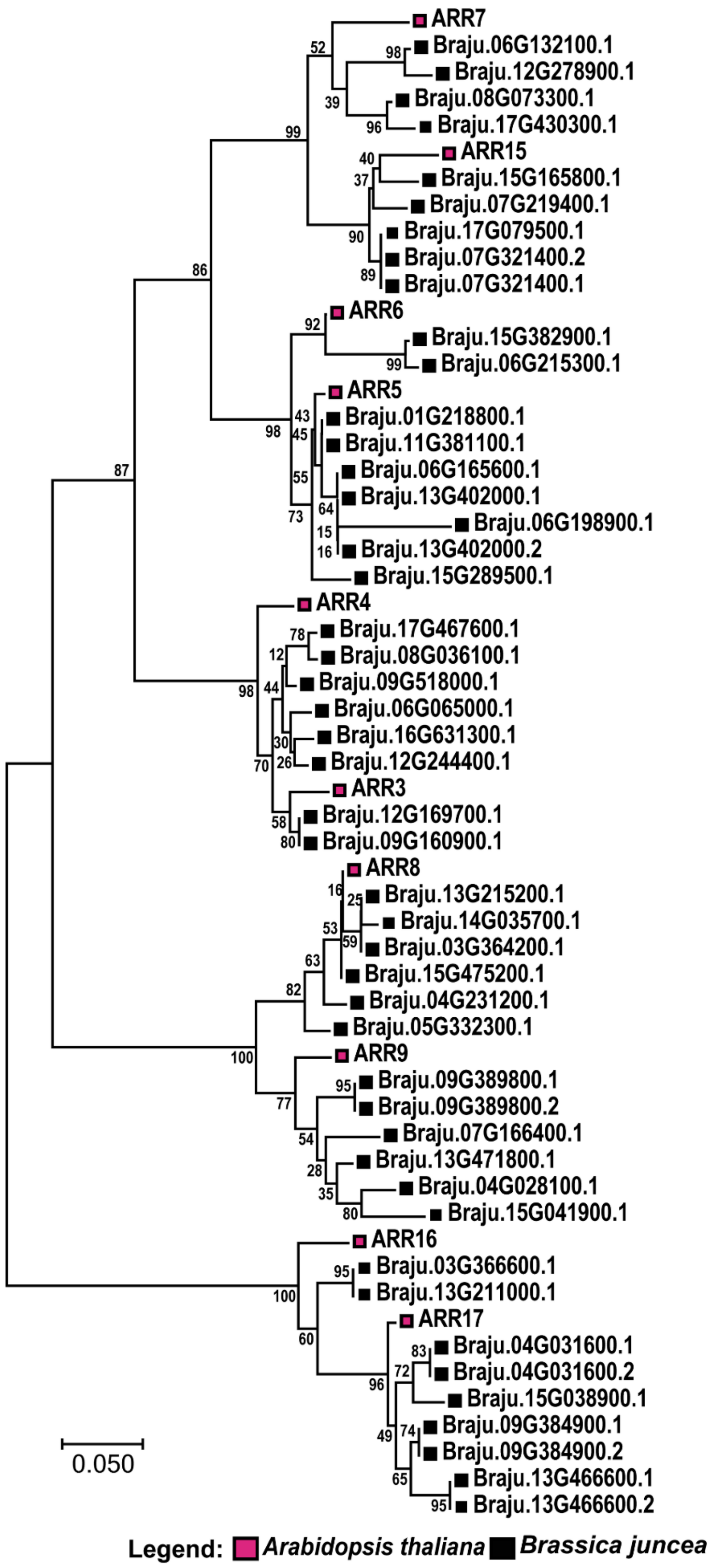

Supplementary Figure S7. Phylogenetic relationship of RRAs in *A. thaliana* and *Brassica juncea*. The unrooted tree is constructed based on the similarity of the RRA protein sequences using the Neighbor-Joining method. The bar indicates the relative divergence of the examined sequences. Individual species are distinguished by the color of the box (dark pink for *A. thaliana*, and black for *Brassica juncea*).

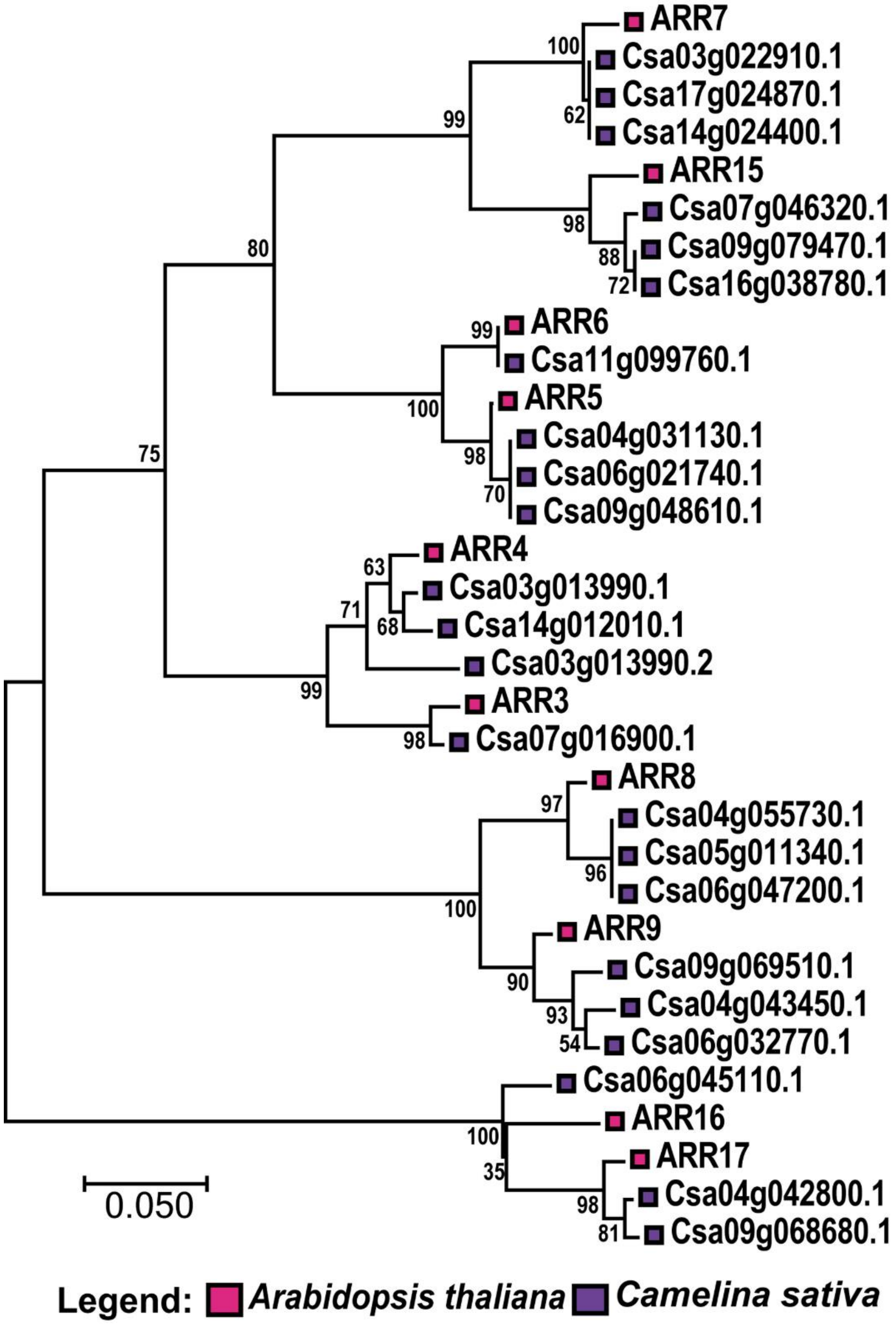

Supplementary Figure S8. Phylogenetic relationship of RRAs in *A. thaliana* and *Camelina sativa.* The unrooted tree is constructed based on the similarity of the RRA protein sequences using the Neighbor-Joining method. The bar indicates the relative divergence of the examined sequences. Individual species are distinguished by the color of the box (dark pink for *A. thaliana*, and dark purple for *Camelina sativa*).
